# Metagenomic, phylogenetic, and functional characterization of predominant endolithic green sulfur bacteria in the coral *Isopora palifera*

**DOI:** 10.1186/s40168-018-0616-z

**Published:** 2019-01-04

**Authors:** Shan-Hua Yang, Kshitij Tandon, Chih-Ying Lu, Naohisa Wada, Chao-Jen Shih, Silver Sung-Yun Hsiao, Wann-Neng Jane, Tzan-Chain Lee, Chi-Ming Yang, Chi-Te Liu, Vianney Denis, Yu-Ting Wu, Li-Ting Wang, Lina Huang, Der-Chuen Lee, Yu-Wei Wu, Hideyuki Yamashiro, Sen-Lin Tang

**Affiliations:** 1 Biodiversity Research Center, Academia Sinica, Taipei, 11529 Taiwan; 2 Tropical Biosphere Research Center, University of the Ryukyus, Okinawa, 905-0227 Japan; 3 Department of Life Science, Tunghai University, Taichung, 40704 Taiwan; 4 Center for Ecology and Environment, Tunghai University, Taichung, 40704 Taiwan; 5 Bioinformatics Program, Institute of Information Science, Taiwan International Graduate Program, Academia Sinica, Taipei, 11529 Taiwan; 6 Institute of Bioinformatics and Structural Biology, National Tsing Hua University, Hsinchu, 30013 Taiwan; 7 Bioresource Collection and Research Center, Food Industry Research and Development Institute, Hsinchu, 30062 Taiwan; 8 Institute of Earth Sciences, Academia Sinica, Taipei, 11529 Taiwan; 9 Institute of Astronomy and Astrophysics, Academia Sinica, Taipei, 11529 Taiwan; 10 Institute of Plant and Microbial Biology, Academia Sinica, Taipei, 11529 Taiwan; 11 Institute of Biotechnology, National Taiwan University, Taipei, 10672 Taiwan; 12 Institute of Oceanography, National Taiwan University, Taipei, 10617 Taiwan; 13 Department of Forestry, National Pingtung University of Science and Technology, Pintung, 91201 Taiwan; 14 Graduate Institute of Biomedical Informatics, College of Medical Science and Technology, Taipei Medical University, Taipei, 11031 Taiwan

**Keywords:** Green sulfur bacteria, Endoliths, *Isopora palifera*, Anaerobic cultivation, Nitrogen fixation

## Abstract

**Background:**

Endolithic microbes in coral skeletons are known to be a nutrient source for the coral host. In addition to aerobic endolithic algae and *Cyanobacteria*, which are usually described in the various corals and form a green layer beneath coral tissues, the anaerobic photoautotrophic green sulfur bacteria (GSB) *Prosthecochloris* is dominant in the skeleton of *Isopora palifera*. However, due to inherent challenges in studying anaerobic microbes in coral skeleton, the reason for its niche preference and function are largely unknown.

**Results:**

This study characterized a diverse and dynamic community of endolithic microbes shaped by the availability of light and oxygen. In addition, anaerobic bacteria isolated from the coral skeleton were cultured for the first time to experimentally clarify the role of these GSB. This characterization includes GSB’s abundance, genetic and genomic profiles, organelle structure, and specific metabolic functions and activity. Our results explain the advantages endolithic GSB receive from living in coral skeletons, the potential metabolic role of a clade of coral-associated *Prosthecochloris* (CAP) in the skeleton, and the nitrogen fixation ability of CAP.

**Conclusion:**

We suggest that the endolithic microbial community in coral skeletons is diverse and dynamic and that light and oxygen are two crucial factors for shaping it. This study is the first to demonstrate the ability of nitrogen uptake by specific coral-associated endolithic bacteria and shed light on the role of endolithic bacteria in coral skeletons.

**Electronic supplementary material:**

The online version of this article (10.1186/s40168-018-0616-z) contains supplementary material, which is available to authorized users.

## Background

All biomes in marine environments, including coral reef ecosystems, are fundamentally dependent on their microbial constituents for biomass and metabolism [1]. Endolithic communities are prevalent within corals, though our understanding of these communities is poorly resolved [2–5]. For example, the endolithic algae contribute more biomass than photosynthetic symbionts in the living corals, indicating that phototrophic endoliths are one of the primary producers in coral reefs [3]. In addition, endolithic microbes are able to contribute to new nitrogen input and process nutrient regeneration in coral reefs [6–8]. For example, diazotrophs process nitrogen fixation in coral skeletons and carbonated sand, adding new nitrogen to the reef at a rate about 5 mg m^2^ day^−1^, which may be essential to the coral reef’s overall nitrogen budget [8].

Endolithic microorganisms are considered major food chain components [9, 10]. Nutrients generated by coral-associated endoliths may be alternative nutrient sources for the coral host [2, 11, 12]. Ferrer and Szmant [13] found that endolithic organisms can fix 55–60% of the nitrogen required by its coral host. In addition, when coral undergoes thermal bleaching, endolithic algae can translocate photosynthetic carbon to their coral host [12].

Endolithic microbes associated with coral skeletons include algae, fungi, bacteria, archaea, and viruses [4, 5]. The green algae *Ostreobium* is the major component of the conspicuous green layers beneath coral tissue in the coral skeleton, which are found in many live corals and are considered a coral symbiont [14]. Recently, it has been suggested that the distribution of *Ostreobium* clades shares similar biogeographical patterns as Symbiodiniaceae [15]. In addition, coral host specificity may also influence endolithic communities because tissue thickness and skeleton structures may result in differences in microenvironments within the coral skeleton [16].

Instead of *Ostreobium* and other aerobic microorganisms, our previous study found that the anaerobic photoautotrophic green sulfur bacteria (GSB) *Prosthecochloris* is dominant and prevalent in the skeleton of the coral *Isopora palifera* [17]. Although GSB are one of the coral-associated bacteria, they are usually present in coral tissue, mucus, and skeleton at relatively low abundances [18–22]. Therefore, the prevalence of *Prosthecochloris* in coral skeletons suggests that abiotic factors, such as oxygen and light intensity, within coral skeletons might be decisive and understudied factors for the composition of endolithic microbes. In addition, GSB are potential nitrogen fixers and photoautotrophs that might act as nitrogen and carbon sources for the coral holobiont [17]. The discovery of the green layers made of predominantly GSB has lead us to reconsider the compositional heterogeneity and diverse functions of endoliths [16, 17]. This phenomenon also raises more in-depth questions about ecological functioning, compositional dynamics, and evolutionary ecology of GSB in corals [16, 17, 22]. However, to date, major gaps still persist in the knowledge of the GSB.

To comprehend GSB’s role within coral skeletons, we conducted multi-level approaches including metagenomics, biochemistry, physiology, histology, and morphology. Using culture-independent and -dependent methods, we discovered putative functions of nitrogen, sulfur, and carbon metabolisms in GSB and other endolithic microbes; visualized the distribution of GSB in coral skeletons; revealed microscopic cellular structures of GSB; and detected nitrogenase activity in GSB. This study is the first, to our knowledge, to use anaerobic cultivation and experiments to characterize the coral microbiota.

## Materials and methods

### Sample collection

Samples of *I. palifera* were collected from Ludao (Green Island), an offshore volcanic islet in the western Pacific Ocean (southeastern Taiwan). Nine healthy coral colonies located at 5–20 m depths were collected from Gongguan (22° 40′ N, 121° 27′ E) on April 21, 2014. The light intensity of sampling locations was 5380–8608 lx and the temperature was 26–27 °C. Coral samples were immediately rinsed twice with sterilized water, then transported to the laboratory (< 1 h) and placed in freezers (− 20 °C). Slurries of green layers were collected from coral skeletons using the method described in Yang et al. [17] and prepared for cell counting (Additional file 1: supplementary materials and methods), 16S rDNA amplicon 454 pyrosequencing, and metagenome analyses.

Three additional coral colonies were collected from the same place on July 25, 2017, for pigment analysis (Additional file1: supplementary materials and methods) and ultra-thin sections and transmission electron microscope observation. Three coral colonies were further collected on October 16, 2017, for anaerobic cultivation of endolithic bacteria.

### DNA extraction

Total genomic DNA of slurry samples from the green layer was extracted using an UltraClean Soil DNA Kit (MioBio, Solana Beach, CA, USA). The DNA extraction followed the manufacture’s protocol with one exception: bacterial cell pellets from the samples of endolithic culture were collected by centrifugation at 7000*×g* at 20 °C for 10 min prior the DNA extraction.

### PCR amplification, 16S rRNA amplicon, metagenomic DNA sequencing and data analyses

To prepare 16S rRNA amplicons, PCR amplification was performed using two universal primers for bacteria—968F (5′-AACGCGAAGAACCTTAC-3′) and 1391R (5′-ACGGGCGGTGWGTRC-3′)—both of which were designed for the bacterial V6–V8 hypervariable regions of the 16S rDNA [23, 24]. The PCR condition and DNA tagging PCR for pyrosequencing condition were the same as those in Yang et al. [17]. A library was prepared and sequenced using the Roche 454 Genome Sequencer Junior system at Genomics Core Lab, Institute of Molecular Biology, Academia Sinica.

For metagenomic analysis, the total genomic DNA was amplified using REPLI-g Mini Kit (QIAGEN) according to the manufacturer’s protocol. All of WGA products were purified using the QIAamp DNA Mini Kit (QIAGEN). All amplified and purified DNAs of the nine samples were sent to Yourgene Bioscience (Taipei, Taiwan) for library preparation and sequencing by Illumina MiSeq system (USA).

Methods for 16S amplicon, metagenome analyses, and draft genome assembly were provided in supplementary material and methods. Summary of 16S amplicon reads and OTUs assigned across samples and metagenome reads and contigs are shown in Table 1. For metagenome analysis, details for sequencing reads summary and gene prediction of metagenomes are described in supplementary data (Additional file 1: Table S1). Bacterial community sequences (SRP154191) and metagenomics reads (SRP151224) were deposited in GeneBank.

**Table 1:** Summary of 16S amplicon reads and OTUs assigned across samples and metagenome reads and contigs

Sample	GIA	GIB	GIC	GID	GIE	GIF	GIG	GIH	GII
16S rRNA gene amplicon data									
Raw reads	1606	1993	658	693	993	2119	1753	2247	2281
Reads without chloroplast and mitochondria	1423	1861	576	616	865	1973	1600	2088	2203
OTUs	59	49	38	28	55	52	34	50	61
Metagenome data									
Total reads of metagenome	4686274	5179522	5764522	6068974	6121690	6943838	4504458	3907868	5426424
Contig number	2190	864	469	3260	403	1777	814	467	1477
Genes (ORF prediction)	13195	6870	1948	17064	587	5998	6133	7155	10033
Genes (with protein length > = 100 aa)	10861	5605	1359	13953	390	4211	4979	5959	8065

Phylogenetic trees were constructed from 16S rDNA and whole genome alignment (with a fragment size set of 200 and step size of 100) of 17 available *Chlorobi* genomes (downloaded from the NCBI Genome database, Additional file 1: Table S2), and the assembled A305 genome was carried out using Gegenees [25]. Detailed methods are provided in the Additional file 1: supplementary materials and methods. 

### Anaerobic endolithic culture

After sampling, coral colonies were immediately placed in an anaerobic jar with an anaerobic pack (Mitsubishi Gas Chemical, Japan) and transferred to an anaerobic chamber within 48 h to collect the green layer. The method for collecting green layers from coral skeletons was the same as described in Yang et al. [17]. However, the entire process was in the anaerobic condition. Endoliths were enriched in the basal medium for *Prosthecochloris* [26], modified by adding glucose (0.05%). Cultures were incubated at 25 °C under bright white light (340 ± 92 lum/ft^2^), under dim light (45.5 ± 31.5 lum/ft^2^), and in dark conditions. After a week, colors appeared in cultures (only dim light) and the cultures were prepared for ultra-thin sections analysis, fluorescence in situ hybridization, pigment analysis, and phylogenetic analysis with V6-V8 16S rDNA sequences.

An anaerobic endolithic culture for nitrogen-fixing functional assays was transferred to the modified basal medium without NH_4_Cl. A subculture was incubated at 25 °C in the dim light condition for a week. Once color appeared in the culture, its subculture was transferred to the modified basal medium without NH_4_Cl again and incubated in the same condition for 2 weeks. Its second subculture was used for assays of acetylene reduction and nanoscale secondary ion mass spectrometry (NanoSIMS). For NanoSIMS, the second subculture was enriched by ^15^N_2_ gas.

### Ultra-thin sections and transmission electron microscope (TEM)

The slurry of green layer and endolithic cultures were centrifuged at 1000 rpm for 5 min to collect cell pellets, then fixed with 2.5% glutaraldehyde and 4% paraformaldehyde, and post-fixed in 1% OsO_4_. The ultra-thin sections (70–90 nm) were stained with 5% uranyl acetate in 50% methanol and 0.4% lead citrate in 0.1 N sodium hydroxide and observed by TEM (A FEI G2 Tecnai Spirit Twin). The detailed protocol was given in the Additional file 1: supplementary materials and methods.

### Fluorescence in situ hybridization (FISH)

Cells of endolithic cultures were fixed, sonicated, and filtered onto polycarbonate membranes (0.2 μm pore size, 25 mm diameter, Whatman). Each filter were divided into two equal squares for two different probe sets of the experiment. FISH was performed using three oligonucleotide probes (EUB338mix [27, 28], GSB532 [29], and Non338 [30]) and observed using a confocal laser scan microscope (LSM 780, Carl Zeiss). The detailed protocol was given in the Additional file 1: supplementary materials and methods.

### Acetylene reduction assay (ARA)

An acetylene reduction assay (ARA) was conducted to detect nitrogenase activity in the GSB-dominant culture [31]. Sterilized vials (100 ml) filled with 80% nitrogen and 20% carbon dioxide gas were sealed with sterilized rubber stoppers. Then, 20 ml of endolithic cultures (the second subculture) were injected (by syringe) into the vials. After adding 10% acetylene to each vial, ethylene was measured after 0, 24, 48, and 96 h by gas chromatography (G-3000, Hitachi, Japan) using Nukol™ Capillary GC Column (size × I.D. 30 m × 0.32 mm, df 0.25 μm, Merck, Germany). To compare nitrogenase activity in endolithic cultures, sterilized endolithic culture were used as negative controls and basal medium as control. Endolithic cultures, negative controls, and controls were incubated in the dim light condition at 27 °C. Differences were tested using *t* test.

### Nanoscale secondary ion mass spectrometry (NanoSIMS)

Cells before and after ^15^N enrichment culture were harvested onto 0.2 μm Au-Pd precoated polycarbonate membrane. FISH experiment described above was performed to label general bacteria by the EUB338 mix probe and GSB by the GSB532 probe. After taking fluorescence images, the membrane samples were fixed by copper tape onto one aluminum stub (2.54 cm diameter) and analyzed by NanoSIMS 50 L (Cameca-Ametek, Gennevilliers, France) housed in Academia Sinica, Taiwan. 0.8–1.2 pA Cs + primary beam was used to raster over the cells of interests. The secondary ions ^12^C^−^, ^12^C^14^N^−^, ^12^C^15^N^−^, ^31^P^−^, and ^32^S^−^ were collected simultaneously by multiple electron multipliers. A 30-μm entrance slit and 350-μm aperture slit were used to reach mass resolving power of 4500. Image data on elements were collected in 50 μm squares at 512 × 512 pixels resolution lasting 1 h. The images and isotope ratio of regions of interest were processed by L’Image software (developed by Larry Nittler, Carnegie Institution of Washington, Washington D.C.).

## Results

### Bacterium-like cell number and composition in the *Isopora palifera* skeleton

**Fig. 1:**
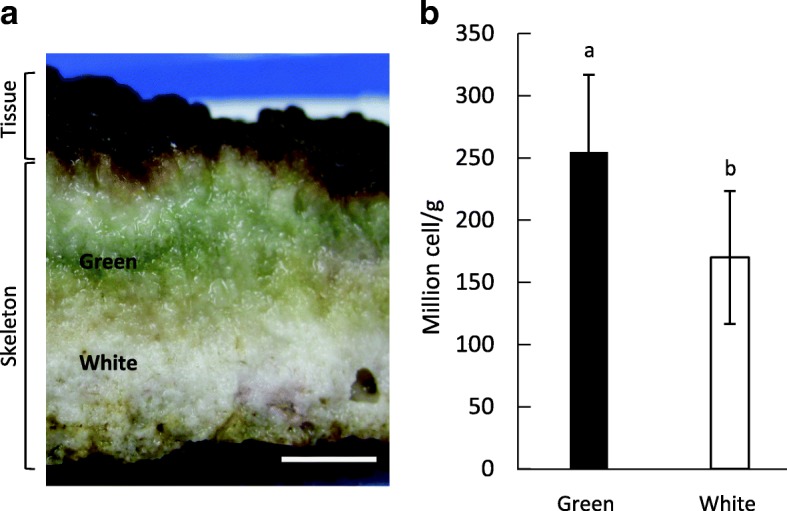
Skeleton of *Isopora palifera* and cell numbers within the skeleton. **a** The green layer was a green color constantly present in the CaCO_3_ skeleton beneath tissue in all colonies of *I. palifera*; the white layer was usual CaCO_3_ skeleton without a green color. The scale bar indicates 1 cm. **b** Average cell numbers from three colonies in the green and white layers. Different marks (**a**, **b**) indicate significant differences in cell number by student’s t-test between the layers (*p* = 0.0068, error bar = standard deviation)

In all of the colonies, the green layer was present in the coral skeleton under coral tissue (Fig. 1a) and there was a significant difference in bacterium-like cell numbers between the green and white layers (Fig. 1b). The green layer had 2.55 × 10^8^ cells/g on average while the white layer had 1.7 × 10^8^ cells/g on average. There were significantly more cells in the green layer than the white layer, with a *p* value of 0.0068 using *t* test (Additional file 1: Figure S1).

**Fig. 2:**
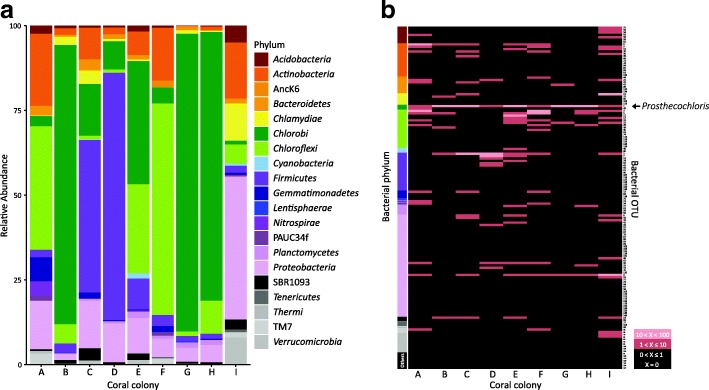
Bacterial taxonomic distribution in the green layer of *Isopora palifera*. **a** Relative abundances of bacteria composition based on 16S rDNA. Colors indicate different bacterial phyla. **b** Heatmap of bacterial OTU abundances in the nine coral colonies. Colors indicating different bacterial phyla are the same as the bacterial phylum in **a** and the detail taxonomic affiliation of OTUs are in the Additional file 1: Table S3. The most dominant OTU1 belongs to the genus *Prosthecochloris* (black arrow) and was present in every colony. The color key indicates the relative abundance of each OTU in each colony

For bacterial composition in the green layer, 16S amplicon results showed that *Chlorobi*, *Firmicutes*, *Chloroflexi*, *Proteobacteria*, and *Actinobacteria* were dominant, and their average relative abundances across all samples colonies were 35.24% (SE 12.40), 15.54% (SE 8.56), 16.58% (SE 7.05), 12.10% (SE 4.05), and 8.29% (SE 2.61), respectively (Fig. 2a). In addition, an OTU (OTU1) belonging to *Chlorobi* (genus *Prosthecochloris*) was found in all samples at relative abundances between 1.16% (colony I) and 87.50% (colony G) (Fig. 2b and Additional file 1: Table S3). In addition, there was no variation in bacterial composition within the green layer along the depths from where colonies were collected (ANOSIM: *R* = − 0.21, *p* = 0.857).

### Putative metabolic pathways of microbes in the *I. palifera* skeleton

According to metagenomics analysis of the green layer, bacteria contributed the most genes in every colony (Additional file 1: Table S4); these genes were predominately from *Chlorobi* in colonies B, C, G, H, and I (Additional file 1: Figure S2), which is comparable to the results from the composition analysis using 454 pyrosequencing for 16S rDNA in colonies B, C, G, and H (Fig. 2a). We identified variation in relative contribution of *Chlorobi* in two sequencing approaches, with low abundance in 16S rDNA colonies A, F, and I and in metagenome colonies A, E, F; this variation can be attributed to different sequencing technologies used and their resolution.

**Fig. 3:**
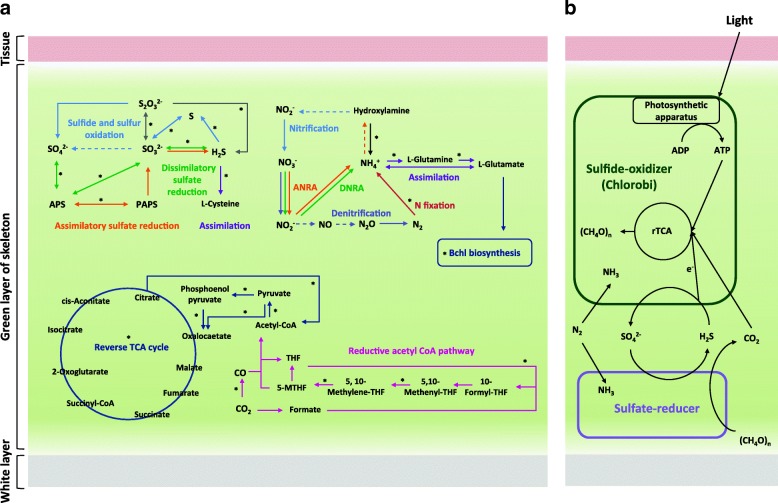
Putative pathways and a proposed syntrophic model of dominant bacteria in the green layer. **a** Putative nitrogen, sulfur, and carbon fixation metabolisms in the green layer. Solid arrows indicate pathways with genes present in the metagenome; dotted arrows indicate pathways with genes not present in the metagenome. Asterisks indicate pathways with genes affiliated to GSB. Colors of arrows indicate different metabolic pathways. **b** A syntrophic model of dominant GSB and sulfate-reducer in the green layer. GSB are able to obtain light that shines into the coral skeleton. During photosynthesis, GSB obtain CO_2_ released by sulfate-reducing bacteria (SRB) and other heterotrophs. For carbon fixed by the rTCA cycle, GSB obtain sulfide as an electron donor, which comes from SRB, while the SRB obtain oxidized sulfur compounds released from GSB. Therefore, GSB and SRB provide sulfur resources for each other. Functional redundancy of nitrogen fixation might be present in the coral skeleton because both GSB and SRB could process nitrogen fixation

Metagenome results showed genes of nitrogen metabolism in the green layer were involved in nitrogen assimilation and reduction pathways of nitrogen fixation, dissimilatory/assimilatory nitrate reduction, and denitrification (Fig. 3, Additional file 1: Figure S3). However, for oxidation pathways, only a pathway involved in nitrification could be identified. Among the genes involved in nitrogen metabolism, genes involved in nitrogen fixation, glutamine/glutamate synthases, and reduction of hydroxylamine were most common and were in turn mainly contributed by GSB. Other bacteria contributing to nitrogen fixation and the reduction of hydroxylamine genes belonged to Firmicutes, while other bacteria contributing glutamine/glutamate synthases genes belonged to *Actinobacteria*, *Bacteroidetes*, *Chloroflexi*, *Proteobacteria*, and *Firmicutes*.

Metagenome analyses focused on sulfur metabolism (Fig. 3, Additional file 1: Figure S4) and showed that GSB, *Firmicutes*, *Chloroflexi*, and *Deltaproteobacteria* contributed complete pathways for assimilatory and dissimilatory sulfur reduction. Especially, GSB contributed all of the pathways of dissimilatory sulfur reduction, including genes for dissimilatory sulfite reductase, APS reductase, and ATP sulfurylase, which are involved in the oxidation of sulfide and sulfite.

The complete reverse TCA (rTCA) cycle and reductive acetyl CoA pathway, which are only present in anaerobes or microaerophiles, were identified in metagenomes (Fig. 3, Additional file 1: Figure S5). For the two pathways, GSB contributed all genes involved in the rTCA cycle, and *Chloroflexi*, *Actinobacteria*, *Nitrospirae*, and GSB contribute genes in partial reactions of the reductive acetyl CoA pathway. Although GSB and other endolithic bacteria also participate in the reductive acetyl CoA pathway, none of them contributed a complete set of genes for this pathway. Therefore, the GSB should be the major contributor to carbon fixation through the rTCA cycle.

### *Candidatus* Prosthecochloris sp. A305 genome recovered through genome binning

Co-assembly of nine metagenomes using Ray-META led to a successful recovery of the *Ca*. Ptc. sp. A305 draft genome after binning the assembled contigs. The draft genome of *Ca*. Ptc. sp. A305 had 75 contigs and was 2,094,032 bp long in total. The longest contig and N50 value were 201,178 and 55,482 bp, respectively. A quality check revealed low contamination (< 1%) in the recovered genome and a moderate to high completeness (~ 78%) estimated by CheckM. The draft genome of *Ca*. Ptc. sp. A305 had 2046 genes, including 2001 protein-coding genes, 24 tRNA genes, and the 47.83% GC content.

Binning results also identified another bin with high completeness, Bin 3. However, this bin has a heterogeneity of 2. Re-binning failed to separate this bin into its sub-bins, which is a bottleneck of the binning algorithms for closely related organisms sharing a bin. Two 16S rRNA copies (1 complete and 1 partial) were present in Bin 3 which were used in 16S rRNA-based phylogenetic analysis, and no further analysis was performed on this bin.

### Anaerobic culture, morphology, and pigment identification of endolithic GSB

**Fig. 4:**
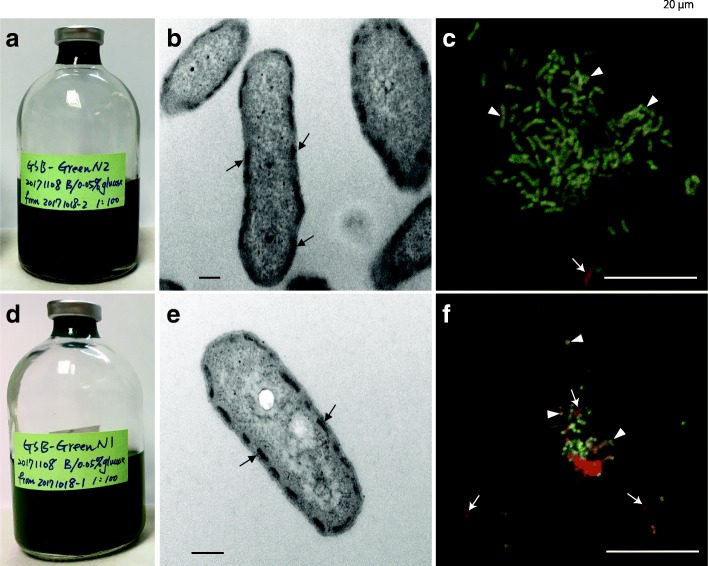
The two endolithic GSB cultures and morphology of the GSB cells. N2 (**a**) and N1 (**d**) cultures grew in the dim light condition and was a brown and green color, respectively. **b**, **e** Photographs of ultra-thin sections of cells from N2 and N1 cultures, respectively, seen through a transmission electron microscope. Most cells in the two cultures have chlorosome-like structures (arrows). Scale bars indicate 200 nm. **c**, **f** Fluorescence in situ hybridization images of cells from N2 and N1 cultures, respectively. GSB cells (arrow heads) are in yellow and other bacteria (arrows) are in red. Scale bars indicate 10 μm

The cultures N2 (brown-green color) (Fig. 4a) and N1 (green color) (Fig. 4d) were obtained from the green layer. Both cultures only grew in the dim light condition (Additional file 1: Figure S6). Furthermore, TEM and FISH were used to identify morphology and ultrastructure of the cells in the culture. Most cells in N2 and N1 were rod-shaped and possessed chlorosome-like structures near their cell membrane (Fig. 4b, e), which is the typical morphology of green sulfur bacteria. In addition, most cells from the skeleton of the green layer and *Ptc. vibrioformis* DSM 260 also had chlorosome-like structures (Additional file 1: Figure S7), which confirmed the hypothesis that cells in the green layer and cultures have the same morphology and ultrastructure. Moreover, FISH images of N2 and N1 also revealed that most cells in the two cultures were GSB (Fig. 4c, f, Additional file 1: Figure S8). The cells in N2 were long-rod while cells of N1 were rod-shaped. Later, upon analyzing the absorption spectrum of N1, *Ptc. vibrioformis* and *Chl. luteolum* that we observed had major peaks in 420–430 nm and 650–660 nm, confirming the presence of Bchl c (Additional file 1: Figure S9a) and supporting the result of the metagenome analysis (Additional file 1: Figure S9b). Nevertheless, N2 also had a maximum peak at 650–660 nm, but the major peak in the short wave was at 460–470 nm, indicating the presence of Bchl e.

### Phylogenetic analysis of GSB from the coral skeleton

**Fig. 5:**
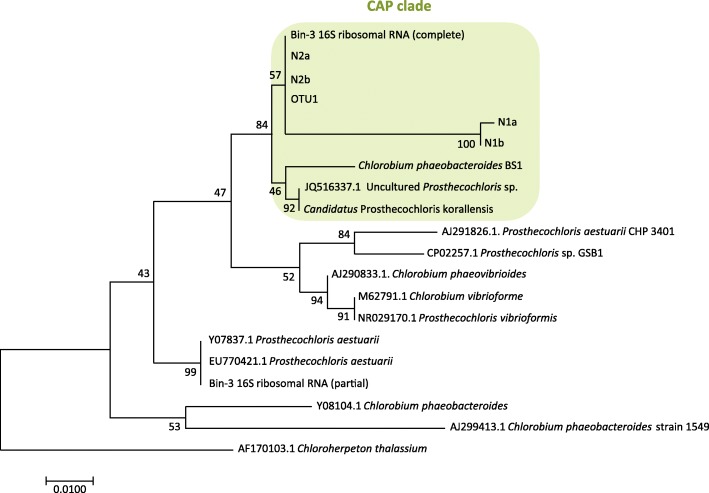
Phylogenetic tree of 16S rDNA from endolithic cultures (N2 and N1), OTU1 and genome bins. The tree was generated using the maximum-likelihood method with 1000 bootstraps. Scale bar denotes 0.01 changes per nucleotide sites. Both sequences from cultures (each culture has two biological repeat), OTU1 and a complete 16S sequence from Bin 3 clustered with sequences of other coral-associated *Prosthecochloris* (CAP), forming the CAP clade

Regarding the identification by V6-V8 of 16S rDNA sequences based on the NCBI database, both sequences from N2 and N1 were closest to a sequence of *Prosthecochloris* sp. (MF423475.1), with similarity more than 98% and 96% ,respectively. Although there was no 16S rDNA gene found in the genome of *Ca*. Ptc. sp. A305, the 16S rDNA derived from Bin 3 had 99% similarity to OTU1 and the 16S rDNA sequence of N2 (Fig. 5). Albeit N1 was not the same as N2, Bin 3, or OTU1; all of them were close to a cluster of coral-associated *Prosthecochloris* (CAP), including *Ca*. Ptc. korallensis [22] (Fig. 5).

In addition, comparing the entire *Ca*. Ptc. sp. A305 genome to other complete and draft genomes of already sequenced species from phylum *Chlorobi*, the phylogeny separated the genomes into three major clades (*Chlorobi*, *Prosthecochloris*, and *Chlorobaculum*) as expected (Additional file 1: Figure S10). *Ca*. Ptc. sp. A305 and Bin 3 were in clade *Prosthecochloris* with the nearest neighbor—*Ca*. Ptc. korallensis—also isolated from coral, forming a CAP clade that is congruent with 16S phylogenetic analysis.

### N fixation of endolithic GSB and H_2_S production by sulfur-reducing bacteria (SRB)

**Fig. 6:**
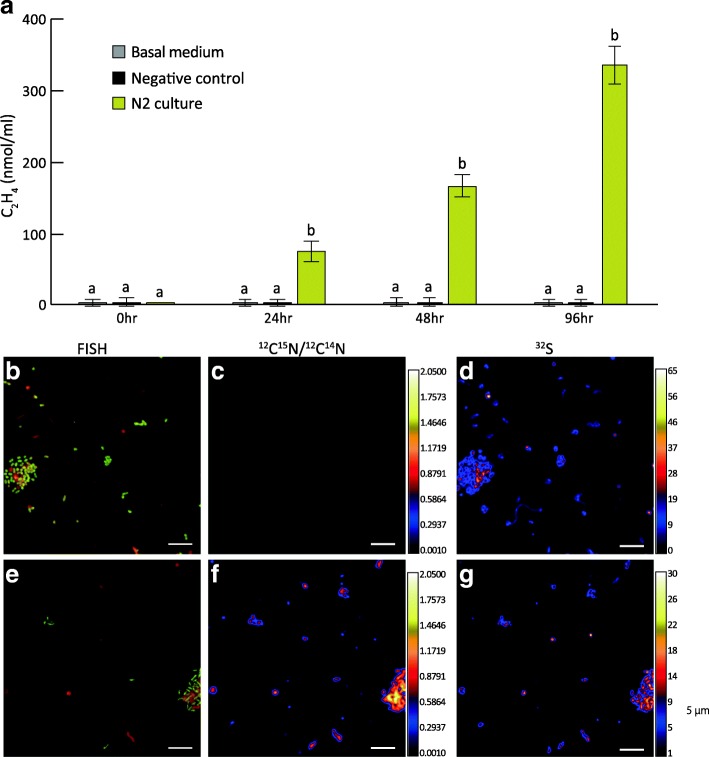
Nitrogen fixation activity of the endolithic culture from the green layer of the coral skeleton. **a** Acetylene reduction assay as proxy for nitrogen fixation activity in endolithic culture N2. Basal medium was used as control and sterilized N2 as negative control. **a**, **b** Indicate significantly difference in the concentration of C_2_H_4_ (*p* < 0.002) production for each time point by *t*-test between N2 and controls. **b** Parallel FISH-NanoSIMS images of endolithic enrichment culture (N2) before (**b**, **c**, **d**) and after (**e**, **f**, **g**) ^15^N_2_ incubation. The FISH results before (**b**) and after (**e**) ^15^N_2_ incubation show the endolithic GSB (green) and other microbes (red). Natural abundance of nitrogen isotopic composition (^12^C^15^N/^12^C^14^N) is 0.00367 and is black in the color bar in both images **c** and **f**. The ^32^S images shows the distribution of all microorganisms before **d** and after **g** ^15^N_2_ incubation. Scale bars indicate 5 μm

Since the V6-V8 of N2’s 16S rDNA sequence was consistent with the metagenome bin and the most dominant OTU in all nine colonies, and within the N2 cultures, the OTU of *Prosthecochloris* was the most dominant (with relative abundance of 64.1%, in Additional file 1: Figure S11), the N2 culture was representative culture, and we used it to validate GSB’s ability to fix nitrogen by ARA and FISH-NanoSIMS. Further, the result of ARA (Fig. 6a) showed significantly higher concentrations of C_2_H_4_ than the control and negative control during 24, 48, and 96 h in N2, confirming that the endolithic GSB cultures had nitrogenase activity. To further consolidate the results, we used FISH-NanoSIMS imaging of N2 cultures grown in conditions with ^15^N_2_ as the only nitrogen source showed more ^15^N in cells, determining that endolithic bacteria fixes nitrogen. FISH identified GSB as the dominant bacteria in N2 able to fix nitrogen (Fig. 6b, Additional file 1: Figure S12).

The potential SRB in the community of N2 were *Halodesulfovibrio*, *Desulfuromusa*, unclassified *Desulfuromonadaceae*, and unclassified *Desulfobacteraceae*, together accounting for ~ 13% of the relative abundance (Additional file 1: Figure S11). In addition, the *dsrA* gene was detected in the N2 culture and two samples of the green layer of *I. palifera* (Additional file 1: Table S5). The ratio of *dsrA* gene to 16S gene in N2 and two samples were 0.0461, 0.0006, and 0.0013, respectively. Furthermore, the functional test of sulfate reduction also relied on the N2 culture. After 10 days of cultivation, the N2 cultures produce 1.339 ppm of H_2_S while OD was 0.649 on average (Additional file 1: Table S6), confirming that it was the SRB that reduced sulfate in the cultures.

## Discussion

This study combines multi-approach results of morphology, metagenomics, pigment identification, and anaerobic culture-based experiments to comprehensively characterize GSB in coral skeletons, including their population abundance, genetic and genomic profiles, organelle structure, and specific metabolic functions and activity. To our knowledge, this is the first time that the role of endoliths has been characterized in coral skeletons.

### GSB in the *Isopora palifera* skeleton

Analyses of cell and gene abundances in this study conclude that GSB is the dominant micro-organism in the *Isopora palifera* skeleton. Special attention should be paid to microenvironmental factors such as light and oxygen availability because they can shape the microbial community composition inside the coral skeleton by restricting the growth of many oxygen- and light-dependent microorganisms. The growth conditions and physiological, cellular, and genomic features of the GSB uncovered in this study are strongly linked to two specific environmental factors: light and oxygen, although other environmental factors may also play a role. The coral skeleton is a harsh environment with extremely low light and oxygen [32]. Most studies have not measured either factors, especially not in the skeleton, probably because it is technically difficult; however, Magusson and co-workers found that less than 0.1–2% of the incident photosynthetically available radiation was reaching the green layer of corals *Montipora monasteriata* and *Porites cylindrica* [33]. This matches with the high density of cells in the green layer and that N1 and N2 cultures can grow exclusively under dim light condition. Oxygen levels are likely low in the skeleton of *I. palifera* as GSB and *Firmicutes*, which are restricted anaerobic bacteria, are present there in high abundance. Lack of oxygen may also be a crucial factor in preventing endolithic algae—which has been reported as the dominant group in studies on other corals—from becoming dominant [14]. Light attenuation is also an essential factor that could make the *I. palifera*’s skeleton a suitable habitat for GSB to thrive. GSB can use the light not only to generate its own energy, but also to become a primary producer for an entire organismic community. Furthermore, because light availability varies in different layers of the skeleton, GSB only thrives at certain depths in the skeleton. However, it is worth mentioning that GSB likely takes advantage of light availability to obtain more energy than other co-existing bacteria that only rely on respiration and fermentation.

Light intensity in the natural environment (sampling site, 5 m to 20 m) is 8608 to 5, 380 lx. However, based on culture conditions, we know that GSB can successfully grow in the light intensity of 45.5 ± 31.5 lum/ft^2^ (489.58 lx), suggesting that the coral skeleton has low-light conditions. The closely related species *Prosthecochloris phaeobacterioides* BS1 (old name: *Chlorobium phaeobacteriodes* BS1), discovered in the Black Sea, is specially adapted to low light, even less than 0.25 μmol photons m^−2^ s^−1^ (13.5 lx) [34]. More physiological tests are needed to detect the range of light intensity in coral skeletons. In addition, GSB are able to live in low-light conditions because of chlorosome, their typical photosynthetic apparatus [35, 36], and bacteriochlorophyll c, d, or e [37–39]. Encircling-type chlorosomes in the GSB cells were evidently detected by TEM in this study. As for the pigment content, the metagenomes showed that Bchl c was major bacteriochlorophyll detectable in the green layer (Additional file 1: Figure S9 b). However, the absorbance spectra of pigment extractions from the brown-green cultures (N2) indicate the existence of Bchl e.

Types of pigments highly associated with environmental adaptation to light absorption and cell growth vary in GSB [40]. This study’s pigment and genomic analyses suggest that GSB should have a specific light absorption spectrum and light sensitivity preferences. The different colors of the cultures also support the differential preference for light sensitivity and spectrum in the endolithic GSB of *I. palifera*.

### New GSB species and coral-associated *Prosthecochloris* (CAP)

The phylogenetic results of 16S rDNA and whole genomes indicate that the dominant endolithic GSB in the green layer are new species of genus *Prosthecochloris*. Both phylogenetic analyses show the endolithic GSB strains (N1 and N2) and two metagenomic bins (*Ca*. Ptc. sp. A305 and Bin 3) clustered close to two species, *Ptc. phaeobacteroides* BS1 (synonym *Chl. phaeobacteroides* BS1) and *Ptc*. *aestuarii* DSM 271. These two species are typical marine representatives of GSB and have the largest phylogenetic distances from other GSB [41]. Interestingly, the GSB strains and bins were clustered with *Ca*. Ptc. korallensis into a single clade, which is also a *Prosthecochloris* species discovered from metagenomes of coral-associated bacteria [22]. Hence, we propose a group of coral-associated *Prosthecochloris* (CAP) based on their phylogenetic distance from other free-living marine *Prosthecochloris* isolates. CAP was proposed to play symbiotic roles in coral holobionts of different coral species [17, 22]. Identifying more members of CAP may facilitate an understanding of symbiotic or ecological relationships between them and their coral hosts, and the evolution of the marine group *Prosthecochloris*.

### The role of GSB in the nutrient cycle

Coral reefs are a net source of fixed nitrogen in oligotrophic environments [42], and nitrogen uptake is important for coral health and the balance of coral holobiont [43]. In the nitrogen cycle, the processes of nitrogen fixation, nitrification, and denitrification have been identified as being associated with corals, and nitrogen-cycling microbes are commonly detected in regular coral-associated microflora [43]. Among the nitrogen-cycling microbes, nitrogen fixation is thought to be proceeded by oxygenic phototrophic bacteria, such as *Cyanobacteria* [44], and anaerobic phototrophic diazotrophs, such as GSB [17]. In this study, We used ARA and FISH-NanoSIMS to provide the first evidence demonstrating that endolithic GSB can fix nitrogen. Therefore, given that coral-associated diazotrophs are related to coral health [43], we suggest that the dominant GSB in coral skeletons plays an essential role in fixing nitrogen in the coral holobiont.

GSB are able to perform anoxygenic photosynthesis with the help of chlorosomes for light harvesting via the rTCA cycle for carbon fixation [45]. One feature of GSB is their ability to obtain electrons by oxidizing sulfide, sulfur, or thiosulfate to support photosynthesis [46, 47]. In the green layer, endolithic GSB acquire electrons from oxidizing sulfide and sulfite rather than thiosulfate, which is similar to the ways that *Chl. tepidum* [45], marine group GSB *Ptc. aestuarii*, and *Ptc. vibrioformis* do it [38].

In some anaerobic systems, such as lake water and microbial mats, a syntrophic association between GSB and sulfur-reducing bacteria (SRB) appears because sulfate produced by GSB is used as an electron acceptor by SRB, and biogenic sulfide produced by SRB is used as an electron donor by GSB [38, 48–50]. In this study, although GSB was dominant in the endolithic cultures, the presence of the *dsrA* gene and production of H_2_S (1.339 ppm) also indicate the existence and function of SRB. Among the endolithic communities in the green layer, *Firmicutes* was the second most dominant phylum, of which class *Clostridia* was present in all of the coral colonies at high abundances. It is known that a large group of SRB is found among *Clostridia* [51]. In addition, *Deltaproteobacteria* that contains most sulfate reducers was also one of the major groups in the endolithic community. Hence, the syntrophic association between GSB and *Firmicutes*/*Deltaproteobacteria* might occur in the coral skeleton. Beyond the function of sulfur reduction, some SRB, including *Firmicutes* and *Deltaproteobacteria*, are nitrogen fixers [52, 53]. *Firmicutes* has been considered a member of the nitrogen-fixing symbionts within the coral holobiont [54]. Hence, we suggest that the relationship between *Firmicutes*/*Deltaproteobacteria* and GSB in nitrogen fixation is functionally redundant. Taken together, we propose a model for the endolithic metabolic pathways in the skeleton of *I. palifera* that shows syntrophic cycling of oxidized and reduced sulfur compounds between the SRB and GSB (Fig. 3b).

### Environmental factors crucial for shaping the endolithic communities

Although it has been suggested that endolithic microbial constitutions may be dynamic due to changes in microbial composition [16], the effect of environmental factors on shaping and restructuring these microbial communities is rarely discussed. We propose that the difference between aerobic microbe (*Ostreobium*/*Cyanobacteria*)- and anaerobic microbe (GSB)-dominating communities in the green layer could be caused by dynamic variations in environmental factors and coral species. Different coral species have different coral skeleton densities, pore sizes, and genetics-based skeleton structures that together build diverse environments in their coral skeleton and, with dynamic environmental conditions, eventually influence and shape the distribution and structure of microbes [55, 56]. *Ostreobium* was usually found in *Porites* such as *P. lutea* [57], *P. lobate* [2], and *P. astreoides* [15], but was not detected in *I. palifera* [17]. The natural skeleton density of *I. palifera* is higher than *Porites* species (Additional file 1: Table S7), which might facilitate low oxygen concentrations within the skeleton and create a better environment for (facultative) anaerobes to reside. Instead, *Porites* provide more aerobic microenvironments in the skeleton for aerobic microbes and at the same time restrict the occupation of anaerobic microbes.

Therefore, we propose that oxygen availability is a key driver in the construction of endolithic microbial compositions for maintaining ecological functions to govern carbon and nitrogen metabolism in coral skeletons. The anaerobic community fixes carbon and nitrogen—anaerobic photoautotrophs fix carbon (e.g., GSB) and anaerobic diazotrophs fix nitrogen (e.g., GSB or *Firmicutes* in colonies B, C, D, G, and H). When there is some oxygen available in the skeleton, facultative anaerobes (e.g., *Chloroflexi*) are the most dominant and perform carbon fixation and *Actinobacteria* and *Proteobacteria* perform nitrogen fixation (e.g., the colonies A, E, F, and I). The coral skeleton contains sufficient oxygen, so algae *Ostreobium* and *Cyanobacteria* would turn into the prevalent microbial species and take charge of fixing carbon or nitrogen.

Microenvironments within the skeleton differ from time to time, so the nature of skeletons is actually heterogeneous in oxygen availability as well as light intensity. The types of microbial communities proposed are likely to co-exist in different niches in the skeleton at the same time. In other words, endolithic microbial communities are likely to be more diverse and dynamic than the conventional belief.

## Conclusion

Recent advances on the role of microbial communities are revolutionizing our traditional view of the coral holobiont’s physiology. This study sheds light on the functional importance of some dominant groups of bacteria by characterizing their role in nutrients, which may be modulated by microenvironmental conditions prevailing within the coral skeleton. Furthermore, this study addresses an unprecedented challenge of culturing bacteria isolated from coral skeletons in the anaerobic environment.

Although *Ostreobium* has been found to be widespread and closely associated with the skeleton of many coral species [15], our study provides the first details on the ecological functions of endolithic GSB in coral skeletons, pointing out the importance of anaerobic behavior when investigating coral holobionts. We also illustrate a stratification of microbial communities along the physiochemical gradients in coral skeletons, which extend our knowledge of different types of microbial mats and their possible relationship with animals.

Current understanding of the coral-microbe interactions that occur in the coral skeleton is still in its infancy, but we already know that coral skeletons are not only the fundamental, basic scaffolds for the reef habitats of many marine organisms, but also essential carbon sources and sinks in reef ecosystems [58–60]. Tambutté et al. [61] showed that lower pH causes increased porosity in the coral skeleton and thus reduces coral skeleton density. The increased porosity in the coral skeleton may change the concentration of oxygen in the microenvironment, potentially shifting endolithic microbial composition and function. It would be beneficial to corals if those bacteria were found to be functionally associated with nutrient or element cycles, particularly for the corals host. Hence, further understanding of the variation in endolithic microbial communities in response to environmental changes and understanding of the nutrient uptake and health of corals are necessary.

## Additional file

Additional file 1: Supplementary materials and methods. (DOCX 2487 kb)
