# Supplementary material for: Metagenomic, phylogenetic, and functional characterization of predominant endolithic green sulfur bacteria in the coral Isopora palifera
Source: Microbiome. 2019 Jan 4;7:3. doi: 10.1186/s40168-018-0616-z (PMC6320609; doi:10.1186/s40168-018-0616-z)
Supplement: Supplementary file 1 — Supplementary materials and methods. (DOCX 2487 kb) [file 40168_2018_616_MOESM1_ESM.docx]

**Metagenomic, phylogenetic and functional characterization of predominant endolithic green sulfur bacteria in the coral *Isopora palifera***

Shan-Hua Yang^1,2,3,4^, Kshitij Tandon^1,5,6^, Chih-Ying Lu^1^, Naohisa Wada^1^, Chao-Jen Shih^7^, Silver Sung-Yun Hsiao^8,9^, Wann-Neng Jane^10^, Tzan-Chain Lee^1^, Chi-Ming Yang^1^, Chi-Te Liu^11^, Vianney Denis^12^, Yu-Ting Wu^13^, Li-Ting Wang^7^, Lina Huang^7^, Der-Chuen Lee^8^, Yu-Wei Wu^14^, Hideyuki Yamashiro^2^, Sen-Lin Tang^1#^

^1^Biodiversity Research Center, Academia Sinica, Taipei 11529, Taiwan

^2^Tropical Biosphere Research Center, University of the Ryukyus, Okinawa 905-0227, Japan

^3^Department of Life Science, Tunghai University, Taichung 40704, Taiwan

^4^Center for Ecology and Environment

^5^Bioinformatics Program, Institute of Information Science, Taiwan International Graduate Program, Academia Sinica, Taipei 11529, Taiwan

^6^Institute of Bioinformatics and Structural Biology, National Tsing Hua University, Hsinchu 30013, Taiwan

^7^Bioresource Collection and Research Center, Food Industry Research and Development Institute, Hsinchu 30062, Taiwan

^8^Institute of Earth Sciences, Academia Sinica, Taipei 11529, Taiwan

^9^Institute of Astronomy and Astrophysics, Academia Sinica, Taipei 11529, Taiwan

^10^Institute of Plant and Microbial Biology, Academia Sinica, Taipei 11529, Taiwan

^11^Institute of Biotechnology, National Taiwan University, Taipei 10672, Taiwan

^12^Institute of Oceanography, National Taiwan University, Taipei 10617, Taiwan

^13^Department of Forestry, National Pingtung University of Science and Technology, Pintung 91201, Taiwan

^14^Graduate Institute of Biomedical Informatics, College of Medical Science and Technology, Taipei Medical University, Taipei 11031, Taiwan

^#^corresponding author

**Keywords:** Green sulfur bacteria, endoliths, *Isopora palifera*, anaerobic cultivation, nitrogen fixation

**Supplementary materials and methods**

**Bacterium-like cell count**

Samples from the green and white layers were collected using a drill, and slurry of coral skeletons were kept in 10 x TE buffer (10 mM Tris-HCl, pH 8.5; 1 mM EDTA, pH 8.0) (<24 h). After removing extra TE buffer, samples were fixed with 2% formaldehyde in 14% EDTA and 2% formaldehyde in 2% acetic acid at 4 °C overnight. Cells were separated from fixed samples using a sonicator (ChromTech, UP-100) with 20 W for 60 s, and washed with 2% acetic acid. Cells were filtered (0.22 μm, Advantec) before being stained with SYBR Green I nucleic acid stain (Invitrogen). Bacterium-like cells were counted on an epifluorescence microscope (Nikon eclipse 90i, Japan). Each sample was counted with fifteen microscope fields of view.

**Data analysis for amplicon 16S rDNA and metagenome datasets**

16S rDNA amplicon sequences generated by Roche 454 GS Junior were sorted into samples by barcodes using an in-house script. Sequences in each sample were screened by MOTHUR [1] to retain those (1) of lengths 350-450 base pairs (bp), (2) with 0 ambiguous bases, and (3) with homopolymers <8 bp. Chimeric sequences detected by UCHIME [2] were also removed. Cross-sample operational taxonomic units (OTUs) were defined at 97% similarity using UPARSE pipeline [3]. Taxonomy of OTUs was predicted by MOTHUR using the implemented Ribosomal Database Project (RDP) Classifier algorithm [4] based on the RDP database. Bacterial community sequences (SRA accession “SRP154191”) were deposited in GenBank (http://ncbi.nlm.nih.gov). Sequencing was performed using the bacterial V6-V8 hypervariable regions of the 16S rDNA, which have higher taxa recovery at the class and phylum levels than the V4 region and has similar taxa recovery in unique and species, genus and family levels to the V4 region [5]. In order to compare studies based on the V4 and V6-V8 regions, the matrices of unweighted UniFrac and Bray-Curtis distance—which represent phylogenetic distance and the abundance of OTUs, respectively—were suggested because of the similar clustering patterns among the V6-V8, V7-V8 and V4 regions [6]. Metagenomic reads obtained from Illumina MiSeq were preprocessed and quality filtered with default parameters in CLC Genomics Workbench (v 6.5.1). Quality filtered and trimmed reads were 251 bp in length. Only paired-end reads were used for individual metagenome de novo assembly using de Bruijn graphs as implemented in CLC Workbench. Assembled contigs in nine metagenomes were used for open reading frame (ORF) prediction with GeneMarkS using MetaGeneMark package [7]. Details for number of reads, contigs, and genes per metagenome are described in supplementary data (Table S1). The metagenomics reads for all the samples were deposited in GeneBank (SRA accession “SRP151224”).

Taxonomic profiling of ORFs was performed by searching ORFs against the NCBI NR database (last downloaded in July 2016) using BLASTX [8] with an e-value of 1e-5, the results of which were imported into MEGAN6 [9] for taxonomy assignment based on matched protein GI number. Taxonomic summaries at the domain and genus levels were performed to identify dominant lineages residing in the metagenomes of the green layer from the coral *Isopora palifera*.

Functional profiling of metagenomes was performed by searching bacterial ORF protein sequences against the KEGG GENES database, genus:prokaryote, with GhostKoala [10]. Further, to understand the role of microbial community in the green layer, carbon fixation, nitrogen metabolism, and sulfur metabolism pathways were analysed at the constituent sub-pathways level. Protein sequences from phylum *Chlorobi* and its constituent genera were also analysed using the same approach to understand the role of green sulfur bacteria (GSB) in carbon, nitrogen, and sulfur metabolisms in the green layer.

Co-assembly and metagenomic binning of quality filtered and trimmed Illumina MiSeq reads from nine metagenomes were performed using Ray-Meta assembler [11] and Maxbin 2.0 (version 2.2.1) [12], respectively. Contigs co-assembled by Ray-meta (with modified kmer of 151 bp) were binned using MaxBin 2.0 with default settings, which utilizes the expectation-maximization approach for binning and recovering draft genomes from metagenomic co-assembled contigs. The resulting bins were then quality checked for completeness and contamination level using CheckM [13], following which a rebinning (running the MaxBin 2.0 binning algorithm again on the primary bins) was performed to remove contamination and outlier contigs. Contigs in the final bins were assigned into taxa using BLASTn against the NCBI NT database.

Phylogenetic analysis was performed using 16S rDNA amplicon sequences and 16S rRNA gene sequences (identified from bins and cultures, respectively). A whole genome alignment (with a fragment size set of 200 and step size of 100) of 17 available *Chlorobi* genomes and the A305 genome (downloaded from the NCBI Genome database, Table S2) were carried out using Gegenees [14], which utilizes a fragment-based alignment algorithm to calculate average similarity among the compared genomes using BLASTn. Average similarity was calculated with a BLAST score threshold of 40%, generating a heatplot matrix that was further used to deduce phylogenetic relationships and visualised in SplitsTree [15]. 16S rRNA gene sequences recovered from Bin-3 (two 16S rRNA sequences), cultures (N1a, N1b, N2a and N2b), and OTU (OTU1) were aligned against the NCBI 16S rRNA database using MUSCLE in MEGA7 [16]. A phylogenetic tree was constructed using the maximum-likelihood (ML) method based on the Jukes-Cantor substitution model and initial tree generation using BioNJ method. The ML tree was visualised after 1000 bootstraps in FigTree [17].

Genome annotation of *Candidatus* Prosthecochloris sp. A305 was performed using Prokka [18] with –locustagA305 –genus Prosthecochloris –kingdom Bacteria –rfam. Rfam option was enabled to identify non-coding RNAs (ncRNAs) with a combination of Infernal and Rfam database.

Functional profiling of *Candidatus* Prosthecochloris sp. A305 was also performed using Ghostkoala to search the KEGG GENES database. Carbon, nitrogen and sulfur metabolism pathways and their constituted genes were analysed for presence in the assembled draft genome.

**Ultra-thin sections and transmission electron microscope (TEM)**

The slurry of green layer and endolithic cultures were centrifuged at 1,000 rpm for 5 min to collect cell pellets, and then fixed with 2.5% glutaraldehyde and 4% paraformaldehyde in 0.1 M phosphate buffer (PB) adjusted to pH 7.0 and kept at room temperature for 1 h. After sonicating using an ultrasonic bath (T-28B, L&R), cells from pellets were rinsed with the PB for 20 min. The cells were post-fixed in 1% OsO_4_ in the PB for 1 h at room temperature, and rinsed with PB for 20 min for three times. After dehydration with alcohol, the cells were embedded in a Spurr’s resin. A Leica Reichert Ultracut S or Leica EM UC6 ultramicrotome was used to make sections. The ultra-thin sections (70-90 nm) were stained with 5% uranyl acetate in 50% methanol and 0.4% lead citrate in 0.1 N sodium hydroxide. A FEI G2 Tecnai Spirit Twin transmission electron microscope at 80 kV was used for viewing and the images are captured with Gatan Orius CCD camera.

**Fluorescence *in situ* hybridization (FISH)**

Cells of endolithic cultures were fixed in 2% paraformaldehyde (16%, EM-grade, Electron Microscopy Sciences) in 1x PB and left at 4 °C overnight, and exchanged 100% ethanol (EtOH) after centrifugation at 13,000 rpm for 30 min. The cells stored in the 100% EtOH were sonicated at 20 W for 30 s (UP-100, B03-Ultrasonic Processor, E-Chrom), filtered onto polycarbonate membranes (0.2 µm pore size, 25 mm diameter, Whatman), and washed twice with 1x PB. Each filter was embedded in 0.1% low-melting-point agarose (Certified Low Melt Agarose, BIO-RAD) to avoid cell loss during the *in situ* hybridization experiment, and divided the filter into two equal squares (around 1 x 0.5 cm^2^) for two different probe sets of the experiment. FISH was performed using three oligonucleotide probes: EUB338mix (5′-GCWGCCWCCCGTAGGWGT-3′) [19, 20] labeled with fluorochrome Cy3 for targeted domain bacteria, GSB532 (5′-TGCCACCCCTGTATC-3′) [21] labeled with Alexa 488 for the green sulfur bacteria and Non338 (5′-ACATCCTACGGGAGGC-3′) [22] labeled with Alexea 488 as negative control. Each filter was covered with hybridization buffer (10% v/v formamide, 0.9 M NaCl, 20 mM Tris-HCl adjusted to pH 8.0 and 0.01% SDS), and an oligonucleotide probe set (EUB338mix&GSB532 for one filter and EUB338mix & Non338 for another filter). The filters were incubated at 45 °C for 3 h and then washed in 15 ml falcon tubes containing preheated wash buffer (0.45 M NaCl, 20 mM Tris-HCl adjusted to pH 8.0 and 0.01% SDS) in a water bath at 48 °C for 15 min. Following washing, the filters were immediately soaked in cold water for 10 s. The filters were air dried and mounted in Citifluor AF1 solution (Citifluor) and observed using a confocal laser scan microscope (LSM 780, Carl Zeiss) using two channels for Cy3 and Alexa 488.

**Pigment analysis**

Pigments were extracted from bacterial cells using 80% acetone at 4 °C for 30 min. Centrifugation of homogenate was performed at 4,400 x g for 10 min at 4 °C, the supernatant was collected for subsequent analyses. Absorption spectra of pigments was analyzed at room temperature using a Hitachi U-2800 Spectrophotometer (Japan). Pigments extracted from *Prosthecochloris* *vibrioformis* DSM 260 and *Chl. luteolum* DSM 273 were used as positive controls.

**Measurement of skeleton density**

Skeletal density (g/cm^3^) was determined by the ratio of mass per volume of each individual coral skeleton. The mass was measured using an electronic analytical balance (BEL M503i, ±0.001g), and the volume (cm^-3^) was estimated using MeshLab software (Consiglio Nazionale delle Ricerche, Italy) from a digitalized 3D object. This model resulted from the fusion of 12 shots of the coral sample scanned at different angles using a HP David SLS-2 structured white light technology scanning system equipped with a 360° turntable (max mesh density: 1,200,000 vertices per scan; resolution/precision up to 0.1% of scan size down to 0.06 mm).

**Quantitative PCR (qPCR) assay for *dsrA* gene**

For the qPCR assay, the primer sets RH1-dsr-F and RH3-dsr-R [23] were used to target the *dsrA* gene. The hypervariable V6 region of the 16S rRNA gene was used as an endogenous control by using primers 967F (5'-CAACGCGAAGAACCTTACC-3') and 1046R (5'-CGACAGCCATGCANCACCT-3') [23]. Quantitative PCR assays were carried out using Applied Biosystems QuantStudio^TM^ 5 Real-Time PCR System. All qPCR programs consisted of 2 min at 50 °C, 2 min at 95 °C and 40 cycles of 15 s at 95 °C and 60 s at 60 °C. These reactions contained 10 μl PowerUp SYBR Green Master Mix (Thermo Fisher Scientific), 3.4 μl of sterilized nuclease-free water, 0.3 μl each of the forward and reverse primers and 1 μl of DNA template. Each sample was performed in triplicate.

**Determination of H_2_S**

The H_2_S from endolithic cultures was measured by gas chromatography (GC4890, Shimadzu, Japan) using HP-5 Column (30 m x 0.53 mm, Agilent, USA) and Flame Photometric Detector (FPD). At the same time, the optical density at 600 nm of the cultures were measured by Beckman Coulter DU 700 Series UV/Vis Spectrophotometers (USA).

**Supplementary Result**

**Density of coral skeletons**

To associate skeleton density with oxygen availability in coral skeletons, skeleton density of *I. palifera* was measured and compared with other coral skeletons. The density of *I. palifera* of this study had higher average value (1.65 g cm^-3^) to the densities in the previous observation (Table S6). Also, comparing *I. palifera* to other coral species that has been reported with endolithic microbes, the density of *I. palifera* had higher average values to the densities of other coral species except for *Goniastrea retiformis* (Table S6).

**Supplementary Reference**

1. Schloss PD, Westcott SL, Ryabin T, Hall JR, Hartmann M, Hollister EB et al. Introducing mothur: open-source, platform-independent, community-supported software for describing and comparing microbial communities. Appl Environ Microbiol. 2009;75:7537–7541.
2. Edgar RC, Haas BJ, Clemente JC, Quince C, Knight R. UCHIME improves sensitivity and speed of chimera detection. Bioinformatics. 2011;27:2194–200.
3. Edgar RC. UPARSE: highly accurate OTU sequences from microbial amplicon reads. Nature Methods. 2013;10:996–998.
4. Wang Q, Garrity GM, Tiedje JM, Cole JR. Naïve Bayesian classifier for rapid assignment of rRNA sequences into the new bacterial taxonomy. Appl Environ Microbiol 2007;73:5261–5267.
5. Yarza P, Yilmaz P, Pruesse E, Glöckner FO, Ludwig W, Schleifer KH, Whitman WB, Euzéby J, Amann R, Rosselló-Móra R. Uniting the classification of cultured and uncultured bacteria and archaea using 16S rRNA gene sequences. Nat Rev Microbiol. 2014;12:635-45.
6. Tremblay J, Singh K, Fern A, Kirton ES, He S, Woyke T, Lee J, Chen F, Dangl JL, Tringe SG. Primer and platform effects on 16S rRNA tag sequencing. Front Microbiol. 2015;6:771.
7. Besemer J, Lomsadze A, Borodovsky M. GeneMarkS: a self-training method for prediction of gene starts in microbial genomes. Implications for finding sequence motifs in regulatory regions. Nucleic Acids Res. 2001;29:2607–2618.
8. Altschul SF, Gish W, Miller W, Myers EW, Lipman DJ. Basic local alignment search tool. J Mol Biol 1990;215:403–410.
9. Huson DH, Beier S, Flade I, Górska A, El-Hadidi M, Mitra S et al. MEGAN community edition - interactive exploration and analysis of large-scale microbiome sequencing data. PLoS Comput Biol. 2016;12:e1004957.
10. Kanehisa M, Sato Y, Morishima K. BlastKOALA and GhostKOALA: KEGG tools for functional characterization of genome and metagenome sequences. J Mol Biol. 2016;428:726–731.
11. Boisvert S, Raymond F, Godzaridis E, Laviolette F, Corbeil J. Ray Meta: Scalable de novo metagenome assembly and profiling. Genome Biol. 2012;13:R122.
12. Wu YW, Simmons BA, Singer SW. MaxBin 2.0: an automated binning algorithm to recover genomes from multiple metagenomic datasets. Bioinformatics. 2015;32:605–607.
13. Parks DH, Imelfort M, Skennerton CT, Hugenholtz P, Tyson GW. CheckM: assessing the quality of microbial genomes recovered from isolates, single cells, and metagenomes. Genome Res. 2015;25:1043–1055.
14. Ågren J, Sundström A, Håfström T, Segerman, B. Gegenees: Fragmented alignment of multiple genomes for determining phylogenomic distances and genetic signatures unique for specified target groups. PLoS One 2012;7:e39107.
15. Huson DH, Bryant D. Application of phylogenetic networks in evolutionary studies. Mol Biol Evol. 2006;23:254–267.
16. Kumar S, Stecher G, Tamura K. MEGA7: molecular evolutionary genetics analysis version 7.0 for Bigger Datasets. Mol Biol Evol. 2016;33:1870–1874.
17. Rambaut A. FigTree v.1.4.2: Tree Figure Drawing Tool. 2012; http://tree.bio.ed.ac.uk/software/figtree.
18. Seemann T. Prokka: Rapid prokaryotic genome annotation. Bioinformatics. 2014;30:2068–2069.
19. Amann RI, Binder BJ, Olson RJ, Chisholm SW, Devereux R, Stahl DA. Combination of 16S rRNA-targeted oligonucleotide probes with flow cytometry for analyzing mixed microbial populations. Appl Environ Microbiol. 1990;56:1919–1925.
20. Daims H, Brühl A, Amann R, Schleifer KH, Wagner M. The domain-specific probe EUB338 is insufficient for the detection of all Bacteria: development and evaluation of a more comprehensive probe set. Syst Appl Microbiol. 1999;22:434–444.
21. Tuschak C, Glaeser J, Overmann J. Specific detection of green sulfur bacteria by in situ hybridization with a fluorescently labeled oligonucleotide probe. Arch Microbiol. 1999;171:265–272.
22. Wallner G, Amann R, Beisker W. Optimizing fluorescent in situ hybridization with rRNA-targeted oligonucleotide probes for flow cytometric identification of microorganisms. Cytometry. 1993;14:136–143.
23. Ben-Dov E, Brenner A, Kushmaro A. Quantification of sulfate-reducing bacteria in industrial wastewater, by real-time polymerase chain reaction (PCR) using *dsrA* and *apsA* genes. Microb Ecol. 2007;54: 439–451.
24. Chen CP, Tseng CH, Chen CA, Tang SL. The dynamics of microbial partnerships in the coral *Isopora palifera*. ISME J. 2011;5:728–740.

**Supplementary Figures**


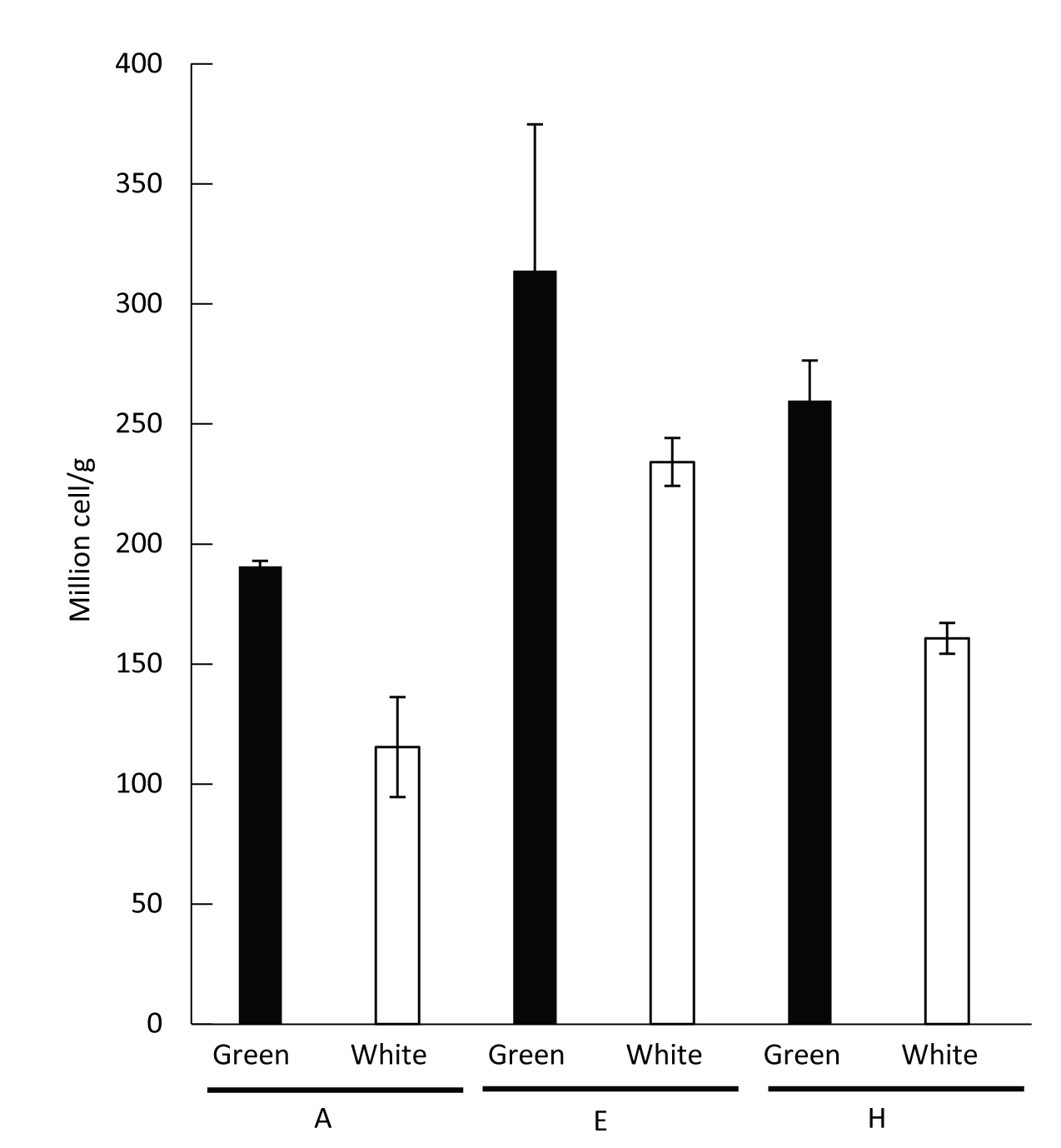


Figure S1 Average cell numbers from samples of colonies A, E and H in the green and white layers of the coral skeleton. Error bar = standard deviation.


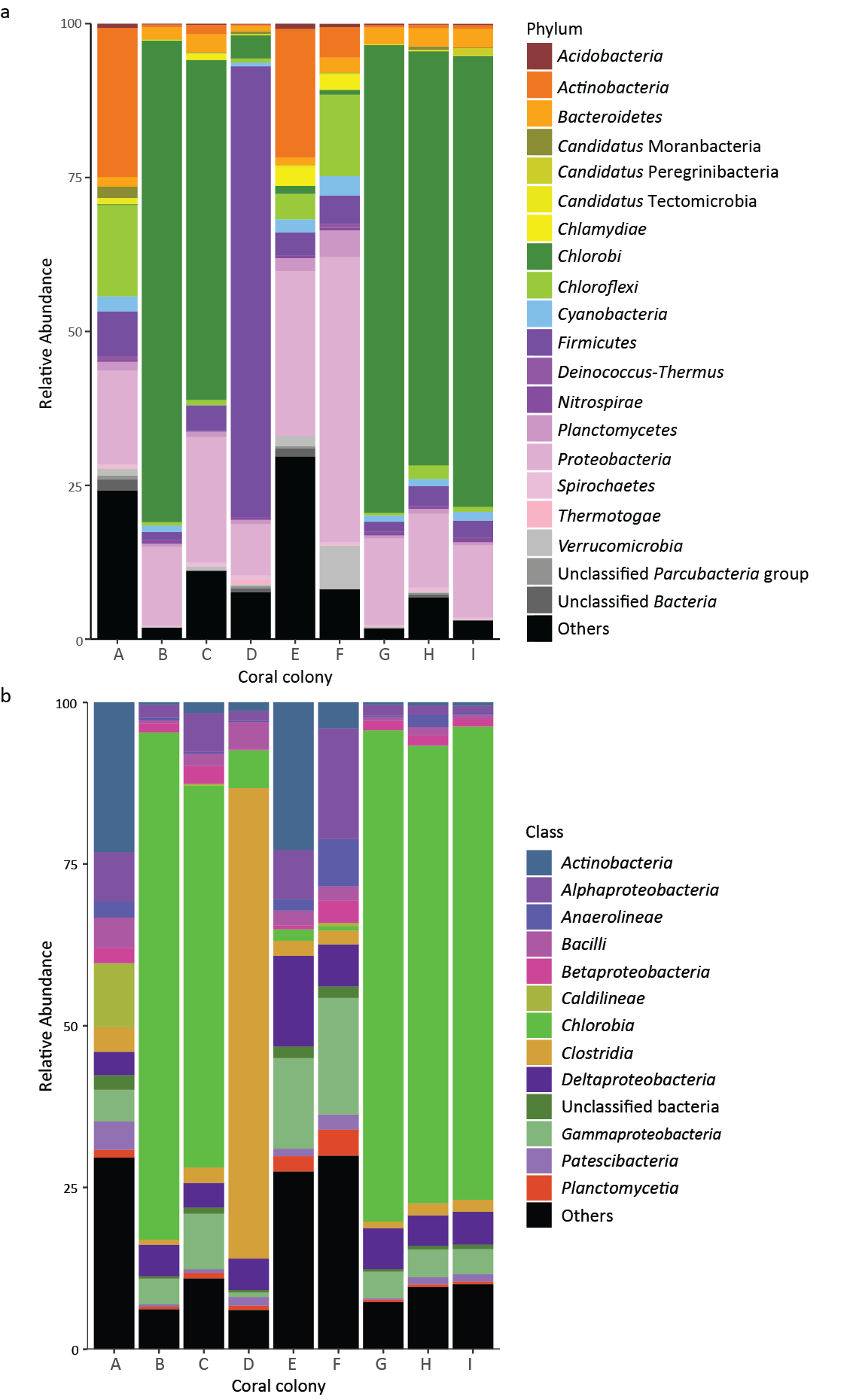


Figure S2 Taxonomic distribution of metagenome genes. (a) Colors indicate bacterial phylum. (b) Colors indicate bacterial class.

| Step | Reaction | Gene | A | B | C | D | E | F | G | H | I |
| --- | --- | --- | --- | --- | --- | --- | --- | --- | --- | --- | --- |
| Dissimilatory nitrate reduction | Nitrate🡪Nitrite | NarGHIJ/NapAB |  |  |  |  |  |  |  |  |  |
|  | Nitrite🡪Ammonia | NirBD/NrfAH |  |  |  |  |  |  |  |  |  |
| Assimilatory nitrate reduction | Nitrate🡪Nitrite | NarB/NR/NasAB |  |  |  |  |  |  |  |  |  |
|  | Nitrite🡪Ammonia | NIT-6/NirA |  |  |  |  |  |  |  |  |  |
| Denitrification | Nitrate🡪Nitrite | NarGHIJ/NapAB |  |  |  |  |  |  |  |  |  |
|  | Nitrite🡪Nitric oxide | NirK/NirS |  |  |  |  |  |  |  |  |  |
|  | Nitric oxide🡪Nitrous oxide | NorBC |  |  |  |  |  |  |  |  |  |
|  | Nitrous oxide🡪Nitrogen | NosZ |  |  |  |  |  |  |  |  |  |
| N_2_ fixation | Nitrogen🡪Ammonia | NifDKH/AnfG |  |  |  |  |  |  |  |  |  |
| Nitrification | Ammonia🡪Hydroxylamine | AmoCAB |  |  |  |  |  |  |  |  |  |
|  | Hydroxylamine🡪Nitrite | Hao |  |  |  |  |  |  |  |  |  |
|  | Nitrite🡪Nitrate | NxrAB |  |  |  |  |  |  |  |  |  |
| Anammox | Nitrite🡪Nitric oxide | NirK/NirS |  |  |  |  |  |  |  |  |  |
|  | Nitric oxide🡪Hydrazine |  |  |  |  |  |  |  |  |  |  |
|  | Ammonia🡪Hydrazine | Hzs |  |  |  |  |  |  |  |  |  |
|  | Hydrazine🡪Nitrogen | Hdh |  |  |  |  |  |  |  |  |  |
| Extracellular | Nitrate🡪Nitrate | Nrt/NrtABCD |  |  |  |  |  |  |  |  |  |
|  | Nitrite🡪Nitrite | Nrt |  |  |  |  |  |  |  |  |  |
| Other | Nitroalkane🡪Nitrite | Ncd2/NAO |  |  |  |  |  |  |  |  |  |
|  | Nitrile🡪Ammonia | nitrilase |  |  |  |  |  |  |  |  |  |
|  | Formamide🡪Ammonia | Formidase |  |  |  |  |  |  |  |  |  |
|  | Ammonia🡪L-Glutamine | GLUL/glnA |  |  |  |  |  |  |  |  |  |
|  | L-Glutamine🡪 L-Glutamate | GLT1/glutamate synthase |  |  |  |  |  |  |  |  |  |
|  | Ammonia🡨🡪L-Glutamate | gudB/rocG gdhA |  |  |  |  |  |  |  |  |  |
|  | Ammonia🡪Carbamoyl-P | CPS1 |  |  |  |  |  |  |  |  |  |
|  | Cabamoyl-P🡪Carbamate | arcC |  |  |  |  |  |  |  |  |  |
|  | CO_2_🡪HCO_3_ | Caronic Anhydrase |  |  |  |  |  |  |  |  |  |
|  | Hydroxylamine🡪Ammonia | hcp |  |  |  |  |  |  |  |  |  |

Figure S3 Nitrogen metabolism pathways in the green layer. Black indicates genes present in the metagenome and green indicates genes in GSB.

| Step | Reaction | Gene | A | B | C | D | E | F | G | H | I |
| --- | --- | --- | --- | --- | --- | --- | --- | --- | --- | --- | --- |
| Assimilatory sulfate reduction | Sulfate🡪APS | PAPSS/Sat/CysND |  |  |  |  |  |  |  |  |  |
|  | APS🡪PAPS | CysC/PAPSS |  |  |  |  |  |  |  |  |  |
|  | PAPS🡪Sulfite | CysH |  |  |  |  |  |  |  |  |  |
|  | Sulfite🡪Sulfide | CysJI/Sir |  |  |  |  |  |  |  |  |  |
| Dissimilatory sulfate reduction and oxidation | Sulfate🡨🡪APS | Sat |  |  |  |  |  |  |  |  |  |
|  | APS🡨🡪Sulfite | AprAB |  |  |  |  |  |  |  |  |  |
|  | Sulfite🡨🡪Sulfide | DsrAB |  |  |  |  |  |  |  |  |  |
| SOX system | Thiosulfate🡪SoxYZ-S-SSO_3_ | SoxA/SoxX |  |  |  |  |  |  |  |  |  |
|  | SoxYZ-S-S-SO_3_🡪Sulfate | SoxB |  |  |  |  |  |  |  |  |  |
|  | SoxYZ-S-S-SO_3_🡪SoxYZ-S-SH | SoxB |  |  |  |  |  |  |  |  |  |
|  | SoxYZ-S-SH🡪SoxYZ-SH |  |  |  |  |  |  |  |  |  |  |
|  | SoxYZ-S-SH🡪SoxYZ-S-SO_3_ | SoxC/SoxD |  |  |  |  |  |  |  |  |  |
|  | SoxYZ-S-SO_3_🡪SoxYZ-SH | SoxB |  |  |  |  |  |  |  |  |  |
| Other sulfur related pathway steps | L-Serine🡪O-Acetyl-L-serine | CysE |  |  |  |  |  |  |  |  |  |
|  | O-Acetyl-L-serine🡪L-cysteine | Cysk/cysO |  |  |  |  |  |  |  |  |  |
|  | Sulfide🡨🡪L-Homocysteine | metB/mccB |  |  |  |  |  |  |  |  |  |
|  | L-Homoserine 🡪O-succinyl-L-homoserine | metA |  |  |  |  |  |  |  |  |  |
|  | O-succinyl-L-homoserine 🡪Succinate | metZ |  |  |  |  |  |  |  |  |  |
|  | Sulfate(ext)🡪sulfate (int) | CysPUWA |  |  |  |  |  |  |  |  |  |
|  | Alkanesulfonate (ext) 🡪Alkanesulfonate(int) | SauACB |  |  |  |  |  |  |  |  |  |
|  | Alkanesulfonate🡪Sulfite | ssuD |  |  |  |  |  |  |  |  |  |
|  | Tetrathionate🡪 Thiosulfate | doxA |  |  |  |  |  |  |  |  |  |
|  | Thiosulfate🡪sulfite | TST/MPST/sseA |  |  |  |  |  |  |  |  |  |
|  | Trithionate🡪Thiosulfate | trithiohydrolase |  |  |  |  |  |  |  |  |  |
|  | Trithionate🡪sulfite | dsrA |  |  |  |  |  |  |  |  |  |
|  | Taurine (ext)🡪Taurine | TauACB |  |  |  |  |  |  |  |  |  |
|  | Taurine🡪Sulfite | tauD |  |  |  |  |  |  |  |  |  |
|  | Thiosulfate🡪Sulfide | phsA,psrA |  |  |  |  |  |  |  |  |  |
|  | Sulfide🡪(Sulfide)_n-1_ | sqr |  |  |  |  |  |  |  |  |  |
|  | Sulfide🡪Sulphur | fccB |  |  |  |  |  |  |  |  |  |
|  | PAPS🡪APS | CysQ |  |  |  |  |  |  |  |  |  |
|  | Sulfite🡪Sulfur | ETHE1 |  |  |  |  |  |  |  |  |  |

Figure S4 Sulfur metabolism pathways in the green layer. Black indicates genes present in the metagenome and green indicates genes in GSB.

| Step | Reaction | Gene | A | B | C | D | E | F | G | H | I |
| --- | --- | --- | --- | --- | --- | --- | --- | --- | --- | --- | --- |
| Reductive Citric acid cycle | Oxaloacetate🡪Malate | mdh |  |  |  |  |  |  |  |  |  |
|  | Malate🡨🡪Fumarate | fumAB |  |  |  |  |  |  |  |  |  |
|  | Fumarate 🡨🡪Succinate | sdhABCD/frdABCDE |  |  |  |  |  |  |  |  |  |
|  | Succinate🡨🡪Succinyl-CoA | sucD/succC/smtA1B |  |  |  |  |  |  |  |  |  |
|  | Succinyl-CoA 🡪  α ketoglutarate | korABCD |  |  |  |  |  |  |  |  |  |
|  | α ketoglutarate🡪Isocitrate | IDH1 |  |  |  |  |  |  |  |  |  |
|  | Isocitrate🡪cis-Aconitase | Aco/acnB |  |  |  |  |  |  |  |  |  |
|  | Cis-Aconitase🡪Citrate | Aco/acnB |  |  |  |  |  |  |  |  |  |
|  | Citrate🡪Acetyl-CoA | ACLY/aclAB |  |  |  |  |  |  |  |  |  |
| Wood-Ljungdahl Pathway | CO_2_🡪Formate | fdhAB |  |  |  |  |  |  |  |  |  |
|  | Formate🡪Formyl-THF | fhs |  |  |  |  |  |  |  |  |  |
|  | Formyl-THF🡪Methenyl-THF | folD |  |  |  |  |  |  |  |  |  |
|  | Methenyl-THF🡪  Methylene-THF | folD |  |  |  |  |  |  |  |  |  |
|  | Methylene-THF🡪  Methyl-tetra-hydrofolate | met-F |  |  |  |  |  |  |  |  |  |
|  | Methyl-tetra-hydrofolate🡪 Tetra-hydrofolate | acs-E |  |  |  |  |  |  |  |  |  |
|  | Tetra-hydrofolate🡪  Formyl-THF | fhs |  |  |  |  |  |  |  |  |  |
|  | Methyl-tetra-hydrofolate🡪 Acetyl-CoA | cdhED |  |  |  |  |  |  |  |  |  |
|  | CO_2_🡪CO | cooS/coxM/coxL |  |  |  |  |  |  |  |  |  |
|  | CO🡪Acetyl-CoA | cdhED |  |  |  |  |  |  |  |  |  |
| Hydroxypropionate-hydroxybutyrate cycle | Acetyl-CoA🡪Malonyl-CoA | accABCD |  |  |  |  |  |  |  |  |  |
|  | Malonyl-CoA🡪  Malonate semialdehyde | mcr |  |  |  |  |  |  |  |  |  |
|  | Malonate semialdehyde 🡪3-Hydroxypropanoate | mcr/malonic semialdehyde redutase |  |  |  |  |  |  |  |  |  |
|  | 3-Hydroxy-propanoate 🡪3-Hydroxypropionyl-CoA | Acrylyl-CoA reductase |  |  |  |  |  |  |  |  |  |
|  | 3-Hydroxypropionyl-CoA🡪 Acryloyl-CoA | Acrylyl-CoA reductase |  |  |  |  |  |  |  |  |  |
|  | Acryloyl-CoA🡪 Propionyl-CoA | Acrylyl-CoA reductase |  |  |  |  |  |  |  |  |  |
|  | Propionyl-CoA🡪 (S)-Methylmalonyl-CoA | Propionyl-CoA- carboxylase |  |  |  |  |  |  |  |  |  |
|  | (S)-Methylmalonyl-CoA🡪 (R)-Methylmalonyl-CoA | MCEE |  |  |  |  |  |  |  |  |  |
|  | (R)-Methylmalonyl-CoA🡪 Succinyl-CoA | MUT |  |  |  |  |  |  |  |  |  |
|  | Succinyl-CoA🡪  Succinate semialdehyde | Succinyl-CoA reductase |  |  |  |  |  |  |  |  |  |
|  | Succinate semialdehyde🡪 4-Hydroxybutanoate | Succinate semialdehyde reductase |  |  |  |  |  |  |  |  |  |
|  | 4-Hydroxybutanoate 🡪4-Hydroxybutanoyl-CoA | 4hbl/4-hydroxybutyrl-CoA synthetase |  |  |  |  |  |  |  |  |  |
|  | 4-Hydroxybutanoyl-CoA🡪 Crotonoyl-CoA | abfD |  |  |  |  |  |  |  |  |  |
|  | Corotonoyl-CoA🡪 (S)-3-Hydroxylbutanoyl-CoA | enoyl-CoA-hydratase |  |  |  |  |  |  |  |  |  |
|  | (S)-3-Hydoxylbutanoryl-CoA🡪 Acetoacetyl-CoA | enoyl-CoA hydratase |  |  |  |  |  |  |  |  |  |
|  | Acetoacetyl-CoA🡪 Acetyl-CoA | atoB |  |  |  |  |  |  |  |  |  |
| Dicarboxylate Hydroxybutyrate Cycle | Acetyl-CoA🡪Malonyl-CoA | accABCD |  |  |  |  |  |  |  |  |  |
|  | Malonyl-CoA🡪  Malonate semialdehyde | mcr |  |  |  |  |  |  |  |  |  |
|  | Malonate semialdehyde 🡪3-Hydroxypropanoate | mcr/malonic semialdehyde redutase |  |  |  |  |  |  |  |  |  |
|  | 3-Hydroxypropanoate 🡪3-Hydroxypropionyl-CoA | Acrylyl-CoA reductase |  |  |  |  |  |  |  |  |  |
|  | 3-Hydroxypropionyl-CoA🡪 Acryloyl-CoA | Acrylyl-CoA reductase |  |  |  |  |  |  |  |  |  |
|  | Acryloyl-CoA🡪Propionyl-CoA | Acrylyl-CoA reductase |  |  |  |  |  |  |  |  |  |
|  | Propionyl-CoA🡪 (S)-Methylmalonyl-CoA | Propionyl-CoA-carboxylase |  |  |  |  |  |  |  |  |  |
|  | (S)-Methylmalonyl-CoA🡪 (R)- Methylmalonyl-CoA | MCEE |  |  |  |  |  |  |  |  |  |
|  | (R)-Methylmalonyl-CoA🡪 Succinyl-CoA | MUT |  |  |  |  |  |  |  |  |  |
|  | Succinyl-CoA🡪  Succinate semialdehyde | Succinyl-CoA reductase |  |  |  |  |  |  |  |  |  |
|  | Succinate semialdehyde🡪 4-Hydroxybutanoate | Succinate semialdehyde reductase |  |  |  |  |  |  |  |  |  |
|  | 4-Hydroxybutanoate 🡪4-Hydroxybutanoyl-CoA | 4hbl/4-hydroxybutyrl-CoA synthetase |  |  |  |  |  |  |  |  |  |
|  | 4-Hydroxybutanoyl-CoA🡪 Crotonoyl-CoA | abfD |  |  |  |  |  |  |  |  |  |
|  | Corotonoyl-CoA🡪 (S)-3-Hydroxylbutanoyl-CoA | enoyl-CoA-hydratase |  |  |  |  |  |  |  |  |  |
|  | (S)-3-Hydoxylbutanoryl-CoA🡪Acetoacetyl-CoA | enoyl-CoA hydratase |  |  |  |  |  |  |  |  |  |
|  | Acetoacetyl-CoA🡪 Acetyl-CoA | atoB |  |  |  |  |  |  |  |  |  |
|  | Acetyl-CoA🡪Pyruvate | pcrABDG |  |  |  |  |  |  |  |  |  |
|  | Pyruvate🡪  Phosphoenolpyruvate | ppdk/pps |  |  |  |  |  |  |  |  |  |
|  | Phosphoenolpyruvate🡪 Oxaloacetate | ppc |  |  |  |  |  |  |  |  |  |
|  | Pyruvate🡨🡪Oxaloacetate | PycAB |  |  |  |  |  |  |  |  |  |
|  | Oxaloacetate🡪Malate | mdh |  |  |  |  |  |  |  |  |  |
|  | Malate🡨🡪Fumarate | fumAB |  |  |  |  |  |  |  |  |  |
|  | Fumarate🡨🡪Succinate | sdhABCD/frdABCDE |  |  |  |  |  |  |  |  |  |
|  | Succinate🡪Succinyl-CoA | sucC/sucD |  |  |  |  |  |  |  |  |  |
| 3-Hydroxypropionate bicycle | Acetyl-CoA🡪Malonyl-CoA | accABCD |  |  |  |  |  |  |  |  |  |
|  | Malonyl-CoA🡪  Malonate semialdehyde | mcr |  |  |  |  |  |  |  |  |  |
|  | Malonate semialdehyde 🡪3-Hydroxypropanoate | mcr/malonic semialdehyde redutase |  |  |  |  |  |  |  |  |  |
|  | 3-Hydroxypropanoate 🡪3-Hydroxypropionyl-CoA | Acrylyl-CoA reductase |  |  |  |  |  |  |  |  |  |
|  | 3-Hydroxypropionyl-CoA🡪  Acryloyl-CoA | Acrylyl-CoA reductase |  |  |  |  |  |  |  |  |  |
|  | Acryloyl-CoA🡪 Propionyl-CoA | Acrylyl-CoA reductase |  |  |  |  |  |  |  |  |  |
|  | Propionyl-CoA🡪(S) - Methylmalonyl-CoA | Propionyl-CoA-carboxylase |  |  |  |  |  |  |  |  |  |
|  | (S)-Methylmalonyl-CoA🡪 (R)- Methylmalonyl-CoA | MCEE |  |  |  |  |  |  |  |  |  |
|  | (R)-Methylmalonyl-CoA🡪 Succinyl-CoA | MUT |  |  |  |  |  |  |  |  |  |
|  | Succinate🡨🡪Succinyl-CoA | smtA1B/sucC/sucD |  |  |  |  |  |  |  |  |  |
|  | Succinate🡨🡪Fumarate | tfrAB/frdABCDE |  |  |  |  |  |  |  |  |  |
|  | Fumarate🡨🡪Malate | fumAB |  |  |  |  |  |  |  |  |  |
|  | Malate🡪L-Malyl-CoA | smtA1B |  |  |  |  |  |  |  |  |  |
|  | L-Malyl-CoA🡪Acetyl-CoA | mcl |  |  |  |  |  |  |  |  |  |
| other | Acetate🡪Acetyl-CoA | ACSS |  |  |  |  |  |  |  |  |  |
|  | Acetate🡨🡪  Acetyl phosphate | ackA |  |  |  |  |  |  |  |  |  |

Figure S5 Metabolism pathways of carbon fixation in the green layer. Black indicates genes present in the metagenome and green indicates genes in GSB.


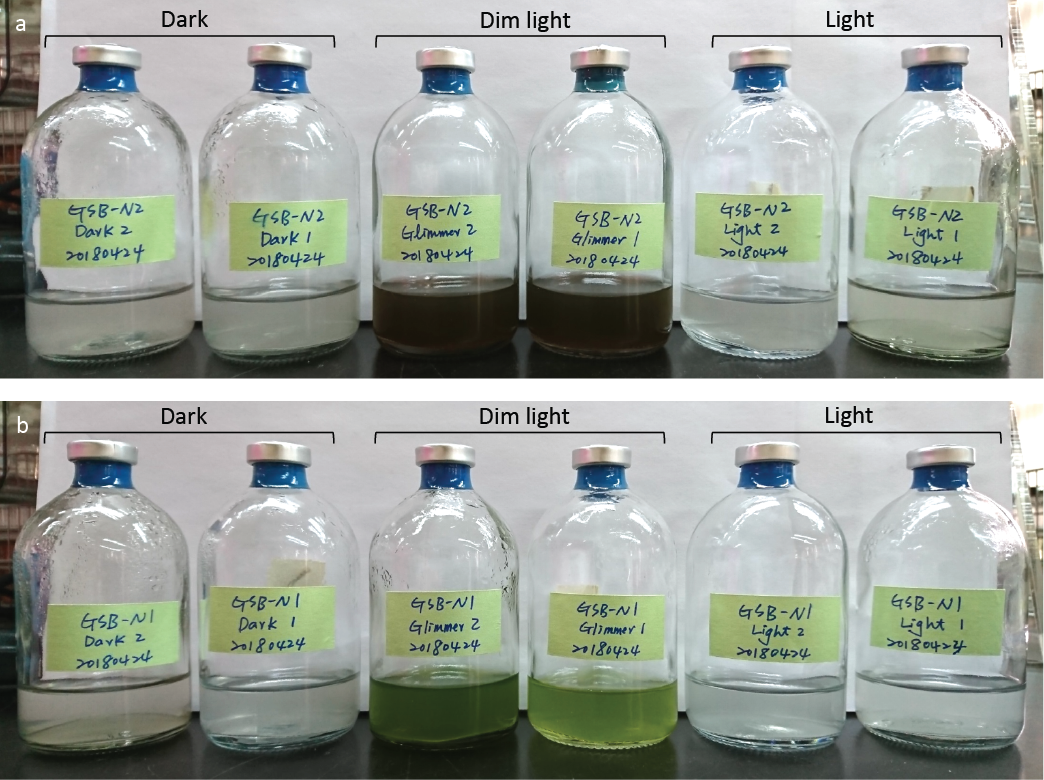


Figure S6 Cultivation of endolithic GSB under different light conditions. (a) N2 and (b) N1 were cultured in dark, dim light (45.5±31.5 lum/ft^2^), and light conditions (340±92 lum/ft^2^) after 6 days of inoculation. For each condition, duplicates of N2 and N1 were cultured; both of N2 and N1 only grew in the dim light condition and were a brown and green color, respectively.


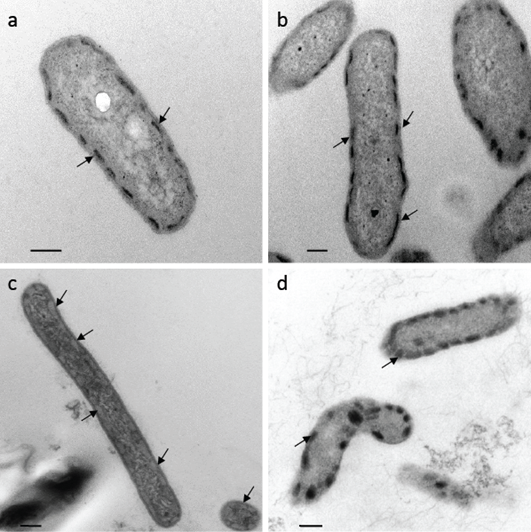


Figure S7 Ultra-thin sections and transmission electronic micrography of microbes from the green layer within skeleton of *Isopora palifera*. (a) and (b) show cells from N1 and N2 cultures; (c) shows cells from the coral skeleton; (d) is cells of *Prosthecochloris vibrioformis* DSM 260. Most cells had chlorosome-like structures (arrows), which are typical structures in GSB. Scale bars indicate 200 nm.


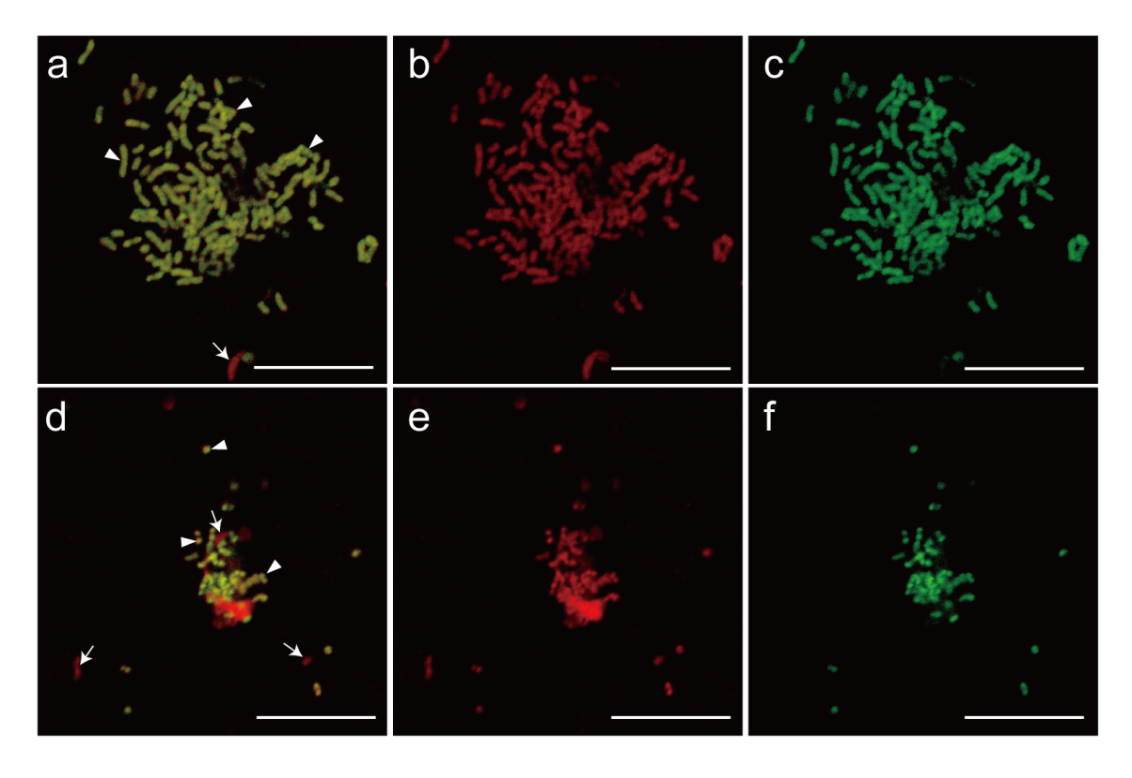


Figure S8 Fluorescence *in situ* hybridization images of endolithic bacterial cultures. (a) and (d) show bacterial composition in brown-green (N2) and green cultures (N1), respectively. GSB cells (arrowheads) are in yellow, and other bacteria (arrows) are in red. GSB was dominant in the two cultures. As showing a reference of the merge images, all bacteria (b and e) and GSB (c and f) in two cultures were detected by EUB338mix probe labeled Cy3 and GSB532 probe labeled Alexa 488, respectively. Scale bars indicate 10 µm.


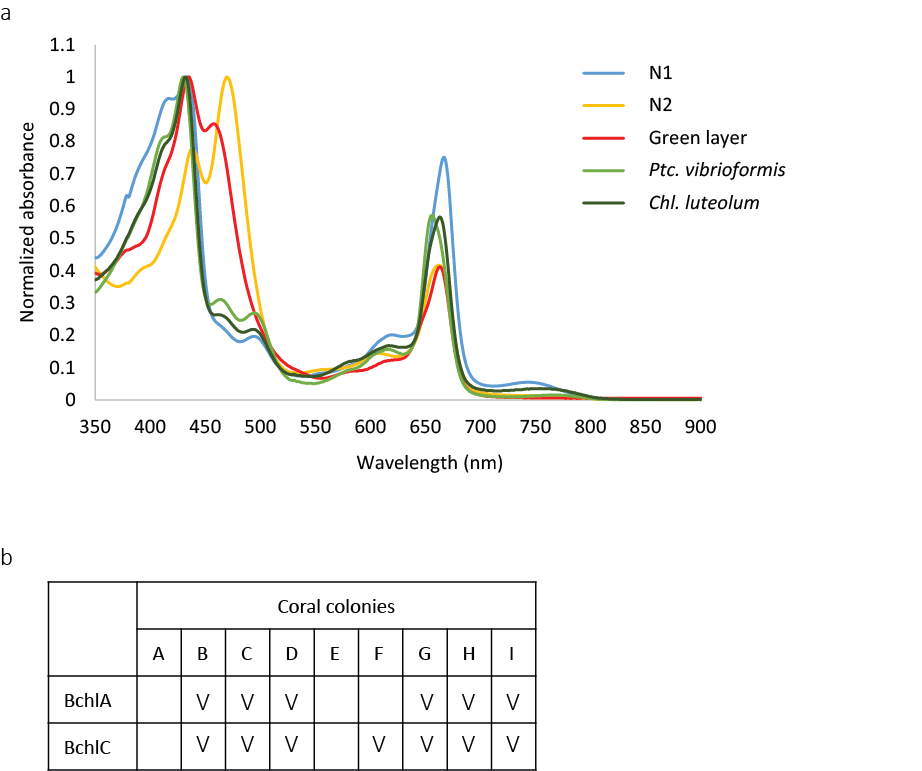


Figure S9 Pigment results from absorbance spectra and metagenome analysis. (a) Absorbance spectra of pigment extractions for the endolithic cultures (N1 and N2), the green layer of coral skeleton, *Ptc. vibrioformis* DSM 260 and *Chl. luteolum* DSM 273 as control. The pigment directly extracted from the skeleton also had the same maxima peaks to that of other samples, but its shortwave peak ranged from 420-460 nm, overlapping with the shortwave peak range in N1 and N2 and indicating that GSB is a factor giving the green layer its color in the skeleton. (b) Presence of bacteriochlorophyll (Bchl) in the nine coral colonies identified by metagenome analysis. “V” indicates the presence of the gene in the metagenome. Only BchlA and BchlC were identified from the metagenomes.


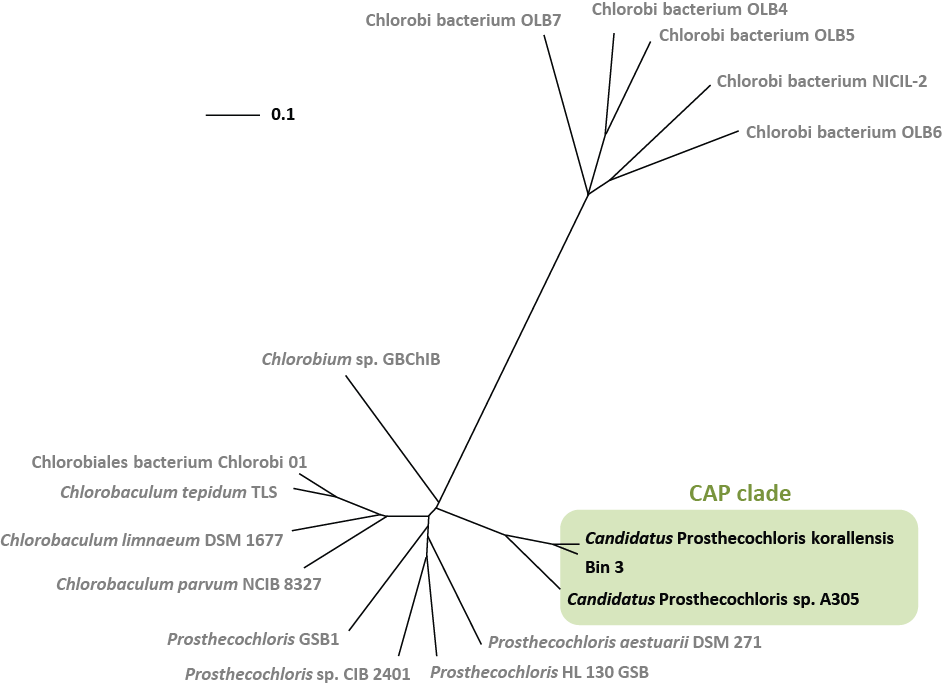


Figure S10 Unrooted whole genome phylogenetic tree constructed with Gegenees v2.1 and visualized using FigTree. *Candidatus* Ptc. A305 and Bin 3 were in the *Prosthecochloris* cluster along with *Candidatus* Ptc. korallensis (isolated from corals). These three genomes form a coral-associated *Prosthecochloris* (CAP) clade.


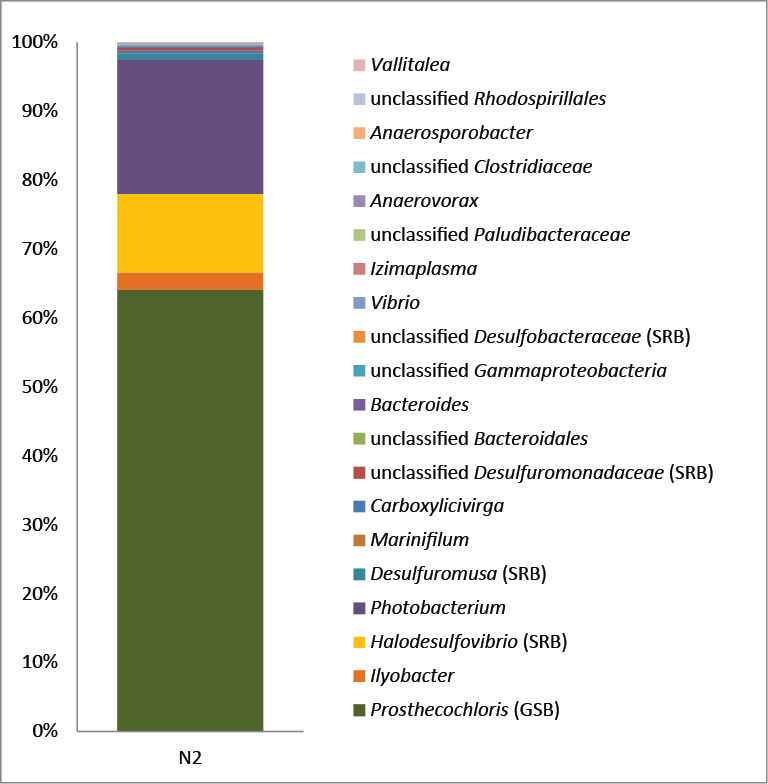


Figure S11 Relative abundance of bacterial OTUs in the N2 culture. Colors indicate bacterial genus of the OTUs. GSB indicates the green sulfur bacteria and SRB indicates the sulfur-reducing bacteria.


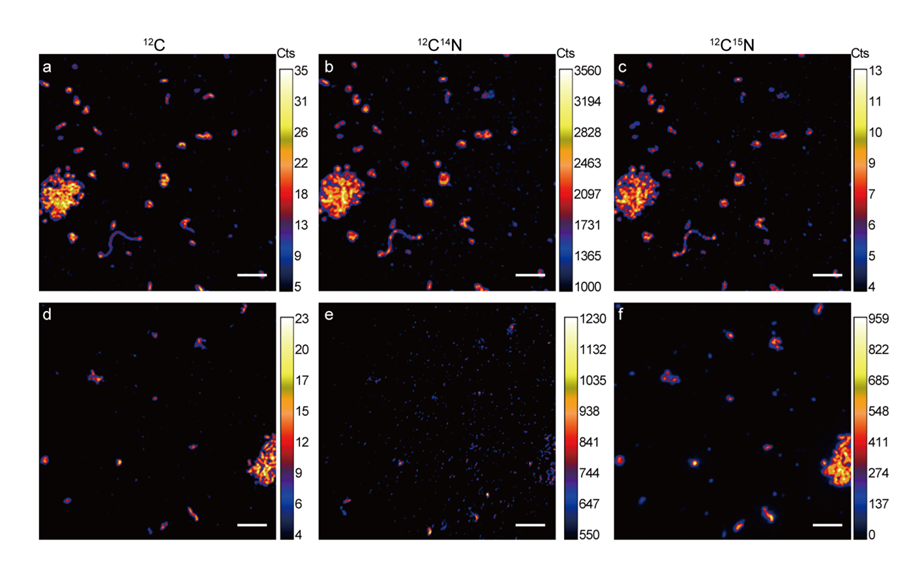


Figure S12 Parallel secondary ion images of ^12^C (a, d), ^12^C^14^N (b, e) and ^12^C^15^N (c, f) for GSB enrichment culture N2 before (a, b, c) and after ^15^N_2_ incubation (d, e, f). Scale bars indicate 5 μm.

**Supplementary Tables**

Table S1. Sequencing reads summary and gene prediction of metagenome

| Sample | GIA | GIB | GIC | GID | GIE | GIF | GIG | GIH | GII |
| --- | --- | --- | --- | --- | --- | --- | --- | --- | --- |
| Total reads | 4686274 | 5179522 | 5764522 | 6068974 | 6121690 | 6943838 | 4504458 | 3907868 | 5426424 |
| Read length | 251 | 251 | 251 | 251 | 251 | 251 | 251 | 251 | 251 |
| Total base | 1176254774 | 1300060022 | 1446895022 | 1523312474 | 1536544190 | 1742903338 | 1130618958 | 980874868 | 1362032424 |
| Total reads after QT | 4673644 | 5170339 | 5754010 | 6053084 | 6104857 | 6931339 | 4467007 | 3902669 | 5412448 |
| Average length after QT | 240.8 | 245.9 | 246.1 | 246.2 | 245.2 | 247.4 | 231.7 | 247.1 | 236.8 |
| Total base after QT | 1125413475 | 1271386360 | 1416061861 | 1490269280 | 1496910936 | 1714813268 | 1035005521 | 964349509 | 1281667686 |
| Contig number | 2190 | 864 | 469 | 3260 | 403 | 1777 | 814 | 467 | 1477 |
| genes (ORF prediction) | 13195 | 6870 | 1948 | 17064 | 587 | 5998 | 6133 | 7155 | 10033 |
| genes (with protein length >= 100 aa) | 10861 | 5605 | 1359 | 13953 | 390 | 4211 | 4979 | 5959 | 8065 |

QT = quality trim (criteria: min length = 35bp, error probability < 0.05)

Table S2 List of Genomes used in the phylogenetic analysis with their Genome size and GC content.

| Genome | Size (Mb) | GC content (%) |
| --- | --- | --- |
| *Candidatus* Ptc. korallensis | 2.58 | 48.3 % |
| *Prosthecochloris aestuarii* DSM 271 | 2.51 | 50.1 % |
| *Prosthecochloris* sp. HL 130 GSB | 2.41 | 52 % |
| *Prosthecochloris* sp. CIB 2401 | 2.39 | 52.1% |
| *Prosthecochloris* sp. GSB1 | 2.46 | 56 % |
| *Chlrobaculum parvum* NCIB 8327 | 2.29 | 55.8 % |
| *Chlorobaculum limnaeum* DSM 1677 | 2.8 | 56.4 % |
| *Chlorobaculum tepidum* TLS | 2.15 | 56.5 % |
| *Chlorobi bacterium* Clorobi_01 | 2.32 | 56.2 % |
| *Chlorobi* sp. GBChlB | 3.06 | 50.9 % |
| *Chlorobi* bacterium NICIL-2 | 2.77 | 56.3 % |
| *Chlorobi* bacterium OLB6 | 2.55 | 49.7 % |
| *Chlorobi* bacterium OLB7 | 3.91 | 57.4 % |
| *Chlorobi* bacterium OLB4 | 2.47 | 37.6 % |
| *Chlorobi* bacterium OLB5 | 3.27 | 37.1 % |

Table S3 Taxonomic affiliation of bacterial OTUs

| OTU | Phylum | Class | OTU | Phylum | Class |
| --- | --- | --- | --- | --- | --- |
| 1 | *Chlorobi* | *Chlorobia* | 41 | *Proteobacteria* | *Alphaproteobacteria* |
| 2 | *Firmicutes* | *Clostridia* | 42 | *Firmicutes* | *Clostridia* |
| 3 | *Chloroflexi* | *Anaerolineae* | 43 | *Proteobacteria* | *Alphaproteobacteria* |
| 4 | *Proteobacteria* | *Gammaproteobacteria* | 44 | AncK6 |  |
| 5 | *Chloroflexi* | *Anaerolineae* | 45 | *Actinobacteria* | *Acidimicrobiia* |
| 6 | *Actinobacteria* | *Acidimicrobiia* | 46 | *Chlorobi* | SJA-28 |
| 7 | *Firmicutes* | *Clostridia* | 47 | *Bacteroidetes* | *Flavobacteriia* |
| 8 | *Proteobacteria* | *Alphaproteobacteria* | 48 | *Proteobacteria* | *Betaproteobacteria* |
| 9 | *Actinobacteria* | *Acidimicrobiia* | 49 | *Cyanobacteria* | 4C0d-2 |
| 10 | *Proteobacteria* | *Deltaproteobacteria* | 50 | TM7 | TM7-3 |
| 11 | *Chlamydiae* | *Chlamydiia* | 51 | *Verrucomicrobia* | *Verrucomicrobiae* |
| 12 | *Chloroflexi* | *Chloroflexi* | 52 | *Proteobacteria* | *Gammaproteobacteria* |
| 13 | *Gemmatimonadetes* | Gemm-2 | 53 | *Proteobacteria* | *Deltaproteobacteria* |
| 14 | SBR1093 | VHS-B5-50 | 54 | *Acidobacteria* | *Acidobacteria-6* |
| 15 | *Actinobacteria* | *Acidimicrobiia* | 55 | *Firmicutes* | *Clostridia* |
| 16 | *Firmicutes* | *Clostridia* | 56 | *Acidobacteria* | PAUC37f |
| 17 | *Chloroflexi* | *Anaerolineae* | 57 | *Proteobacteria* | *Alphaproteobacteria* |
| 18 | *Verrucomicrobia* | *Verrucomicrobiae* | 58 | *Acidobacteria* | *Solibacteres* |
| 19 | *Chloroflexi* | TK17 | 59 | *Acidobacteria* | BPC102 |
| 20 | *Actinobacteria* | *Acidimicrobiia* | 60 | *Chlamydiae* | *Chlamydiia* |
| 21 | *Bacteroidetes* | *Cytophagia* | 61 | *Firmicutes* | *Clostridia* |
| 22 | *Chloroflexi* | *Thermomicrobia* | 62 | *Planctomycetes* | C6 |
| 23 | *Nitrospirae* | *Nitrospira* | 63 | *Chloroflexi* | TK17 |
| 24 | *Firmicutes* | *Clostridia* | 64 | *Verrucomicrobia* | *Spartobacteria* |
| 25 | *Proteobacteria* | *Deltaproteobacteria* | 65 | *Proteobacteria* | *Betaproteobacteria* |
| 26 | *Bacteroidetes* | *Rhodothermi* | 66 | *Proteobacteria* | *Alphaproteobacteria* |
| 27 | *Planctomycetes* | *Phycisphaerae* | 67 | *Proteobacteria* | *Alphaproteobacteria* |
| 28 | TM7 | TM7-1 | 68 | *Thermi* | *Deinococci* |
| 29 | *Chloroflexi* | *Ktedonobacteria* | 69 | *Planctomycetes* | *Planctomycetia* |
| 30 | *Actinobacteria* | *Actinobacteria* | 70 | *Proteobacteria* | *Deltaproteobacteria* |
| 31 | *Acidobacteria* | Sva0725 | 71 | *Proteobacteria* | *Gammaproteobacteria* |
| 32 | *Chlamydiae* | *Chlamydiia* | 72 | *Actinobacteria* | *Actinobacteria* |
| 33 | *Proteobacteria* | *Alphaproteobacteria* | 73 | *Proteobacteria* | *Gammaproteobacteria* |
| 34 | PAUC34f |  | 74 | *Chloroflexi* | *Anaerolineae* |
| 35 | *Proteobacteria* | *Alphaproteobacteria* | 75 | *Verrucomicrobia* | *Verrucomicrobiae* |
| 36 | *Acidobacteria* | AT-s2-57 | 76 | *Lentisphaerae* | *Lentisphaeria* |
| 37 | *Firmicutes* | *Clostridia* | 77 | *Tenericutes* | *Mollicutes* |
| 38 | *Chloroflexi* | *Anaerolineae* | 78 | *Actinobacteria* | *Actinobacteria* |
| 39 | *Actinobacteria* | *Actinobacteria* | 79 | *Proteobacteria* | *Alphaproteobacteria* |
| 40 | *Chloroflexi* | SHA-26 | 80 | *Cyanobacteria* | 4C0d-2 |

Table S3 Taxonomic affiliation of bacterial OTUs (continued)

| OTU | Phylum | Class | OTU | Phylum | Class |
| --- | --- | --- | --- | --- | --- |
| 81 | *Acidobacteria* | Acidobacteria-6 | 121 | *Proteobacteria* | *Alphaproteobacteria* |
| 82 | *Verrucomicrobia* | Verruco-5 | 122 | *Proteobacteria* | *Gammaproteobacteria* |
| 83 | *Gemmatimonadetes* | Gemm-4 | 123 | *Chloroflexi* | Ellin6529 |
| 84 | *Actinobacteria* | *Actinobacteria* | 124 | *Spirochaetes* | *Spirochaetes* |
| 85 | *Actinobacteria* | *Actinobacteria* | 125 | *Chloroflexi* | *Anaerolineae* |
| 86 | *Actinobacteria* | *Actinobacteria* | 126 | *Chlamydiae* | *Chlamydiia* |
| 87 | *Proteobacteria* | *Alphaproteobacteria* | 127 | TM6 | SBRH58 |
| 88 | *Proteobacteria* | *Alphaproteobacteria* | 128 | *Proteobacteria* | *Alphaproteobacteria* |
| 89 | *Bacteroidetes* | *Cytophagia* | 129 | *Proteobacteria* | *Gammaproteobacteria* |
| 90 | *Firmicutes* | *Bacilli* | 130 | OP11 | OP11-3 |
| 91 | WS2 | SHA-109 | 131 | *Proteobacteria* | *Alphaproteobacteria* |
| 92 | *Actinobacteria* | *Actinobacteria* | 132 | *Proteobacteria* | *Gammaproteobacteria* |
| 93 | *Proteobacteria* | *Alphaproteobacteria* | 133 | *Firmicutes* | *Bacilli* |
| 94 | *Proteobacteria* | *Gammaproteobacteria* | 134 | *Proteobacteria* | *Gammaproteobacteria* |
| 95 | *Planctomycetes* | OM190 | 135 | *Proteobacteria* | *Gammaproteobacteria* |
| 96 | *Proteobacteria* | *Alphaproteobacteria* | 136 | *Chloroflexi* | *Ktedonobacteria* |
| 97 | *Proteobacteria* | *Gammaproteobacteria* | 137 | *Bacteroidetes* | *Bacteroidia* |
| 98 | *Proteobacteria* | *Gammaproteobacteria* | 138 | *Gemmatimonadetes* | Gemm-1 |
| 99 | SBR1093 | EC214 | 139 | *Bacteroidetes* | *Cytophagia* |
| 100 | NKB19 |  | 140 | *Proteobacteria* | *Gammaproteobacteria* |
| 101 | *Spirochaetes* | *Leptospirae* | 141 | *Actinobacteria* | *Thermoleophilia* |
| 102 | *Verrucomicrobia* | *Verrucomicrobiae* | 142 | *Firmicutes* | *Bacilli* |
| 103 | *Tenericutes* | *Mollicutes* | 143 | *Proteobacteria* | *Gammaproteobacteria* |
| 104 | *Verrucomicrobia* | *Spartobacteria* | 144 | *Firmicutes* | *Bacilli* |
| 105 | *Proteobacteria* | *Alphaproteobacteria* |  |  |  |
| 106 | *Proteobacteria* | *Gammaproteobacteria* |  |  |  |
| 107 | *Firmicutes* | *Clostridia* |  |  |  |
| 108 | *Proteobacteria* | *Alphaproteobacteria* |  |  |  |
| 109 | *Chlamydiae* | *Chlamydiia* |  |  |  |
| 110 | *Proteobacteria* | *Gammaproteobacteria* |  |  |  |
| 111 | *Proteobacteria* | *Gammaproteobacteria* |  |  |  |
| 112 | *Verrucomicrobia* | *Opitutae* |  |  |  |
| 113 | *Firmicutes* | *Clostridia* |  |  |  |
| 114 | *Chloroflexi* | SAR202 |  |  |  |
| 115 | *Chloroflexi* | *Anaerolineae* |  |  |  |
| 116 | *Proteobacteria* | *Gammaproteobacteria* |  |  |  |
| 117 | *Firmicutes* | *Clostridia* |  |  |  |
| 118 | NKB19 | SHAB590 |  |  |  |
| 119 | *Firmicutes* | *Bacilli* |  |  |  |
| 120 | *Proteobacteria* | *Deltaproteobacteria* |  |  |  |

Table S4. Taxonomic distribution at the domain level of metagenome genes number

| Coral colony | A | B | C | D | E | F | G | H | I |
| --- | --- | --- | --- | --- | --- | --- | --- | --- | --- |
| Bacteria | 8502 | 5154 | 1052 | 13387 | 171 | 3251 | 5228 | 5745 | 8125 |
| Archaea | 1818 | 34 | 12 | 71 | 1 | 49 | 35 | 61 | 106 |
| Eukarya | 115 | 17 | 113 | 129 | 35 | 86 | 6 | 73 | 22 |
| Viruses | 62 | 342 | 45 | 35 | 31 | 136 | 101 | 153 | 72 |
| Unclassified | 37 | 8 | 0 | 14 | 1 | 6 | 3 | 5 | 2 |

Table S5 Ratio of *dsrA* gene to 16S gene in the N2 culture and samples of the green layer of *I. palifera*.

| Sample | dsr Cт | 16S Cт | ΔCт^b^ | Ratio^c^ |
| --- | --- | --- | --- | --- |
| N2 | 17.727 | 13.288 | 4.439 | 0.0461 |
| GN2-1^a^ | 34.129 | 23.428 | 10.701 | 0.0006 |
| GN3-1^a^ | 31.750 | 22.152 | 9.599 | 0.0013 |

^a^samples from the green layer of *I. palifera*

^b^ΔCт = 16S Cт - dsr Cт

^c^R = 2^–ΔCт^

Table S6 Production of H_2_S of the N2 culture

| Sample | OD 600^a^ | H_2_S (ppm) |
| --- | --- | --- |
| N2-1 | 0.654 | 1.431 |
| N2-2 | 0.648 | 1.555 |
| N2-3 | 0.645 | 1.033 |

^a^sample measured at a wavelength of 600 nm

Table S7. Skeleton density of different corals

| Species | Density range (g cm^-3^) | Average  (g cm^-3^) | Source |
| --- | --- | --- | --- |
| *Isopora palifera* (n=9) | 1.49-1.78 | 1.65 | This study |
| *Isopora palifera* (n=6) | ND | 1.52 | Coral trait database* |
| *Isopora palifera* (n=27) | ND | 1.62 | Anderson et al. 2017 |
| *Porites lobata* (data=46) | 1.05-1.62 | 1.38 | Coral trait database |
| *Porites lutea* (data=41) | 1.03-1.87 | 1.28 | Coral trait database |
| *Dipsastraea pallida* (data=1, n=90) | ND | 1.43 | Coral trait database |
| *Goniastrea retiformis* (data=1, n=10) | ND | 1.7 | Coral trait database |
| *Pocillopora damicornis* (n=108) | ND | 1.21 | Anderson et al. 2017 |

*Coral trait database: https://coraltraits.org/ (Madin et al. 2016)

Madin JS, Anderson KD, Andreasen MH, Bridge TCL, Cairns SD, Connolly SR et al. The Coral Trait Database, a curated database of trait information for coral species from the global oceans. Sci Data 2016;3:160017.
